# *Escherichia coli* O157:H7 prevalence in Upper Egypt: impacts on food safety and human Health, with a protection trial using natural antibacterial *Piper cubeba*

**DOI:** 10.1007/s11274-025-04620-3

**Published:** 2025-11-13

**Authors:** Nady Khairy Elbarbary, Nasreddin R. Rhouma, Mostafa M. Abdelhafeez, Ahmed Maher, Hoda S. Sherkawy, Heba M. Nageeb, Ghada Hadad, Sohaila Fathi El-Hawary, Walaa S. Hassan, Wageh S. Darwish, Eman K. Abo Shaala

**Affiliations:** 1 Food Hygiene and Control Department, Faculty of Veterinary Medicine, Aswan University, P.O. Box 81528, Aswan, Egypt; 2 Biology Department, Faculty of Science, Misurata University, P.O. Box 2478, Misurata, Libya; 3 Public Health Department, Faculty of Health Sciences, Alasmarya Islamic University, P.O. Box 495-471, Zliten, Libya; 4 Animal Medicine Department (Infectious Diseases), Faculty of Veterinary Medicine, New Valley University, P.O. Box 72511, El-Kharga, Egypt; 5 Medical Biochemistry Department, Faculty of Medicine, Aswan University, P.O. Box 81528, Aswan, Egypt; 6 Medical Microbiology & Immunology Department, Faculty of Medicine, Aswan University, P.O. Box 81528, Aswan, Egypt; 7 Department of Animal Hygiene and Zoonoses, Faculty of Veterinary Medicine, University of Sadat City, P.O. Box 32958, Sadat City, Egypt; 8 Biology Department, College of Science, Jazan University, P.O. Box 114, Jazan, 45142 Kingdom of Saudi Arabia; 9 Veterinary Teaching Hospital, Faculty of Veterinary Medicine, Assiut University, P.O. Box 71526, Assiut, Egypt; 10 Food Hygiene, Safety, and Technology Department, Faculty of Veterinary Medicine, Zagazig University, Zagazig, Egypt; 11 Food Hygiene Department, Faculty of Veterinary Medicine, Alexandria University, P.O. Box 22758, Alexandria, Egypt

**Keywords:** *E. coli* O157, H7, Toxin, Antibiotic resistance, Virulence factors, *Piper cubeba Linn*

## Abstract

*Escherichia coli* O157:H7 has been linked to intestinal infections in humans and is thought to be a major cause of foodborne illnesses. The research is providing an update on the prevalence and toxin profile of multidrug-resistant *E. coli* O157:H7 in Upper Egypt, from November 2023 to February 2024. A total of 410 samples were obtained from food products, animals, water, and human stool for isolation, identification, and serotyping of multidrug-resistant *E. coli* O157:H7 by using traditional and molecular techniques. Furthermore, the antibacterial effect of *Piper cubeba Linn.* (PcL) extract against *E. coli* O157:H7 was studied in vitro. *E. coli* and *E. coli* O157:H7 were found to have a prevalence of 28% and 8%, respectively, with the highest occurrence in human stool. Depending on serology, 12 different *E. coli* serotypes were identified in the overall samples. The PCR showed that the most virulence genes found were *Stx1* (42.4%), followed by *uidA* (39.4%), *eaeA* (36.4%), *hlyA* (30.3%), and *Stx2* (15.2%). Furthermore, *blaTEM* (42.4%), *tetA* (36.4%), and *Aada1* (27.3%) are the most common resistance genes. Most strains were resistant to ampicillin, penicillin G, and tetracycline (100% each), while florfenicol and ciprofloxacin were entirely sensitive. Furthermore, all concentrations of PcL extract (0.5%, 1%, 3%, and 5%) confirmed significant inhibitory activities against *E. coli* O157:H7 with MIC values of 0.43 mg/mL to 0.74 mg/mL and MBC values of 0.86 mg/mL to 1.76 mg/mL. These results could assist in preserving human health by increasing awareness of the dangers posed by this bacterium and lowering the prevalence of illnesses linked to *E. coli* O157:H7. This study proposes that PcL extract could be employed as a natural antimicrobial additive in food products, which creates new opportunities for its advancement and application in functional foods.

**Supplementary information:**

The online version contains supplementary material available at 10.1007/s11274-025-04620-3.

## Introduction

*Escherichia coli* (*E. coli*) is a facultative, anaerobic, Gram-negative bacillus under the genus Escherichia and the family Enterobacteriaceae. About 90% of *E. coli* strains are commensals that persist in the guts of humans and warm-blooded animals. Their presence in food or drink, however, may indicate improper handling and insufficient washing. *E. coli* can result in serious illnesses, from invasive extra-intestinal problems like sepsis and bacteremia to intestinal infections like diarrhea and dysentery (Altaie et al. 2025). Numerous studies have investigated the prevalence and pathogenic potential of *E. coli* in various food supplies, water sources, and human or environmental samples across Upper Egyptian localities. In Qena City, *E. coli* was found in 80% of beef minced meat, 72.5% of beef sausage, 62.5% of beef kofta, and 67.5% of beef burgers (Dandrawy et al. 2025). In Aswan City, Farag et al. (2023) isolated *E. coli* at a prevalence of 40%, 20%, 20%, and 16% in fresh meat, minced meat, raw milk, and karish cheese, while the prevalence of *E. coli* in humans was 48%. In addition, Karmi (2019) reported that the prevalence of *E. coli* O157:H7 versus other *E. coli* serotypes was 14.3% vs. 85.7% in various meat products from Aswan City.

Pathogenic *E. coli* strains can produce illness by encoding specific virulence features, unlike commensal bacteria (Omar et al. 2025). These strains are categorized into two types depending on the presence of virulence features and the clinical signs of the host: intestinal pathogenic *E. coli* (InPEC) or diarrheagenic *E. coli* (DEC) and extra-intestinal pathogenic *E. coli* (ExPEC) (Denamur et al. 2021). The intestinal pathogenic *E. coli* (InPEC) strains can be subdivided into six groups, including enterohemorrhagic *E. coli* (EHEC) or Shiga toxin-producing *E. coli* (STEC), enterotoxigenic *E. coli* (ETEC), enteropathogenic *E. coli* (EPEC), enteroinvasive *E. coli* (EIEC), diffusely adherent *E. coli* (DAEC), and enteroaggregative *E. coli* (EAEC). The extra-intestinal pathogenic *E. coli* (ExPEC) includes necrotoxigenic *E. coli* (NTEC), uropathogenic *E. coli* (UPEC), and neonatal meningitis *E. coli* (NMEC) (Lee et al. 2023; Omar et al. 2025). Serogroups O26, O45, O91, O103, O111, O113, O121, O128, O145, and O157 are the main types of *E. coli* that can produce Shiga toxins; these toxins are encoded by the *stx1* and *stx2* genes, respectively, and are the main causes of the symptoms (Detzner et al. 2021). Shiga toxin inhibits protein formation in endothelial and other cells, mainly causing thrombotic lesions and disseminated intravascular coagulation, which play a role in gastroenteritis, bloody diarrhea, and uremic syndrome in infected individuals (Younis et al. 2021). Other virulence factors increase the pathogenicity of STEC, including the Intimin (*eaeA*) gene, which is accountable for epithelial cell attachment, adhesion, and aggregation (Younis et al. 2021). Plasmid-encoded enterohemolysin (*hlyA*) encodes for a cytolysin, which can lyse red blood cells and free iron to assist *E. coli* metabolism (Makhado et al. 2022).

The EHEC serotype O157:H7 strains are a significant area of study, not only because they have been associated with global outbreaks, but also due to their low infectious dosage and their ability to persist in extra-intestinal settings. Moreover, O157 types typically carry virulence genes directly linked to EHEC, highlighting their pathogenicity. The H7 antigen (encoded by *fliCH7*) is another important feature of EHEC O157. Human infections with *E. coli* O157:H7 are often linked to the consumption of contaminated animal products, such as raw or undercooked meat, meat products, raw milk, and unpasteurized dairy products (Altaie et al. 2025). In addition to the potential for transmission from person to person, improper storage practices, such as failing to separate raw and ready-to-eat food or improperly managing temperature, might encourage bacterial development (Pakdel et al. 2023).

Antimicrobial resistance is currently a time-sensitive issue for human health, as it is an outcome of the excessive use of antibiotics for therapy of human diseases and livestock production in developing countries (Nady et al. 2024). Studies from different areas have found that *E. coli* O157 is becoming more resistant to frequently used antibiotics (Altaie et al. 2025; Haile et al. 2022). This rise is linked to the spread of resistance genes, which have been occurring in *E. coli* from humans, animals, and the environment (Younis et al. 2021).

Utilizing natural substances to combat spoiling and harmful bacteria has emerged as a key food technology tactic nowadays, when an increasing proportion of consumers oppose the use of artificial antimicrobial agents as food preservatives. To that goal, extensive research has been conducted to identify natural compounds with high bactericidal activity (Gomez-Llorente et al. 2025). Aromatic and medicinal herbs have been employed for therapeutic and cosmetic purposes (Drissi et al. 2022). *Piper cubeba Linn* (PcL) is a plant in the Piperaceae family and Piper genus and a main source of pepper among spices (Zainalabidin and Rukayadi 2019). The plant is valuable for its dried berries, which have been documented to possess various nutritional and pharmacological properties (Drissi et al. 2022). It is a reliable source of essential nutrients, including potassium and iron (Zainalabidin and Rukayadi 2019). It is a source of beneficial phytochemicals, including amides, alkaloids (piperine and cubebin), flavones, flavanones, lignans, neolignans, sesquiterpenes, terpenes, and propenylphenols (Zainalabidin and Rukayadi 2019). In addition, it possesses pharmacological properties, including anti-inflammatory, antibacterial, antioxidant, anti-carcinogenic, and anti-ulcer therapeutic effects. The metabolites and byproducts of the plant demonstrate bacteriostatic and bactericidal impact through a variety of actions, such as the inhibition of bacterial growth, the disruption of the cell membrane, and the attachment to the bacterial DNA (Pradeep 2021).

This research looked into the prevalence, toxin gene detection, and antibacterial resistance patterns of *E. coli* O157:H7 in food products, animals, water, and humans in Aswan, Egypt, as well as the effectiveness of PcL as a natural preservative against *E. coli* O157:H7. These results will increase public awareness and inform the development of efficient control and prevention strategies.

## Methods

### Study area and design

The research was carried out in Aswan Governorate (24°5’20.1768’’ N and 32°53’59.3880’’ E), Egypt, which experiences the warmest and hottest summer in southern Egypt. Aswan is a renowned historical site in Egypt, situated 890 km south of Cairo, and has a total size of 62,726 km², with 62% being plain and 38% hilly. It is the third most popular tourist destination in Egypt, after Cairo and Luxor. In 2024, the Egyptian Central Agency for Public Mobilization and Statistics reported that approximately 1.6 million people were living in the Aswan metropolitan area (Elbarbary et al. 2024).

The investigation was carried out between November 2023 and February 2024 to evaluate the occurrence of *E. coli* O157 in food products, animals, water, and humans. In this research area, the investigated population consisted of healthy-looking animals that were managed under a traditional/extensive husbandry system. Fecal samples were taken via rectal swab from every animal to assess the apparent presence of the target bacteria. Food products (meat and milk) from several retail shops were purchased in their original packaging under various brand names. Water samples were collected from three distinct locations along Nasser Lake and the River Nile. Human samples were gathered from a range of healthcare facilities in Aswan, such as private clinics, hospitals, and diagnostic centers, where individuals with gastrointestinal issues and diarrhea were present. This diversified sampling strategy provides an equitable distribution of the population and increases the dependability of the findings. The gender, age, and residence of every patient were recorded.

### Sampling

Four hundred and ten samples were obtained for bacteriological examination from Aswan Governorate, Egypt, as follows:

### Food products

One hundred and twenty samples in total—twenty each of minced beef, beef kofta, beef burger, raw cow milk, Karish cheese, and yogurt—of different brands (100 g of every product and 100 mL of every liquid sample) were collected in their original packaging from various meat and dairy product retail shops.

### Animal fecal samples

One hundred animal fecal samples in total—thirty each of ox and cow feces and twenty each of sheep and goat feces—were obtained from different farms using sterile cotton swabs. Each rectal swab was directly put in a sterile collection tube holding ten milliliters of buffer peptone water.

### Water samples

Sixty water samples were collected in a sterile multifunctional sample-collecting bottle; thirty samples (100 mL) were taken from each of Nasser Lake and the River Nile. All samples were collected manually following standard procedures and precautions to avoid any extraneous contamination during collection, processing, and transportation. The bottle should be submerged facing upstream, about 20 cm deep, and then turned to collect the sample, avoiding surface debris. Maintain a small air gap at the top of the bottle after filling and immediately replace the cap. Samples were transported to the laboratory on ice pack and preserved in the laboratory at 4 °C until analysis.

### Human stool specimens

A total of one hundred and thirty stool samples were collected from different people under appropriate medical supervision, including 90 patients with diarrhea (45 males and 45 females) and 40 healthy individuals (20 males and 20 females) from both rural and urban areas, aged between one and sixty years, from various healthcare facilities. Every sample was taken in a sterile sample-collecting bottle with ten milliliters of buffer peptone water. This study was conducted in agreement with the guidelines of the Declaration of Helsinki and its later amendments.

All the studied samples were collected and stored in an icebox after being transmitted to the laboratory of the Food Hygiene, Faculty of Veterinary Medicine, Aswan University, for bacteriological examination.

### Bacterial isolation and identification

Ten mL of every liquid sample or 10 g of every product was mixed with 90 mL of tryptic soy broth (TSB; M2059, HiMedia) and kept at 37 °C for 18 h (Quinn et al. 2002). One mL of the test sample was mixed with 9 mL of sterile peptone water (M028S, HiMedia) and kept at 37 °C for 24 h. For selective enrichment, the pre-enriched samples were spread onto MacConkey agar plates (CM0007, Oxoid) and kept for 24 h at 37 °C. The likely pink, rod-shaped *E. coli* colonies were transferred to eosin methylene blue agar (EMB; CM0069, Oxoid) and kept at 37 °C for 24 h. *E. coli* were recognized as dark-centred and flat colonies with a metallic shine. Tests like Gram stain and various biochemical tests (IMViC reaction, catalase, indole production, methyl red, Voges-Proskauer, L-lysine decarboxylation test, citrate use test, and triple sugar iron) were done on the suspected *E. coli* colonies.

To identify the *E. coli* O157 strain from other strains, the detected colonies were sub-cultured on sorbitol MacConkey agar (SMA; CM0813, Oxoid) plates, accompanied with cefixime-tellurite (CT-SMAC) at 37 °C for 24 h. Sorbitol fermenters (pinkish colonies) were identified as non-*E. coli* O157 strains, whereas non-sorbitol-fermenting isolates (colourless or pale colonies) were most likely strains of *E. coli* O157 (Abebe et al. 2023).

### Serotyping of *E. coli*

Positive *E. coli* isolates were serogrouped through an agglutination technique (Edward and Ewing 1972; Zaki et al. 2024) by using commercially available *E. coli* antisera Kit (Denka Kit, Denka Seiken Co., Ltd., Tokyo, Japan), which included 8 polyvalent sera and 43 monovalent sera.

### DNA extraction

The GeneJET™ Genomic DNA Purification Kit (K0722, Thermo Fisher) was employed to isolate DNA from a freshly grown bacterial culture, adhering to the manufacturer’s recommended protocol. DNA purity was measured at 260 and 280 nm using a NanoDrop Spectrophotometer (Thermo Fisher Scientific, USA).

### Verification of *E. coli* O157:H7 by PCR

All non-sorbitol fermenting *E. coli* isolates underwent PCR investigation for the presence of the *rfbEO157* gene and *fliCH7* (Tayh et al. 2022). The process involved: denaturation at 94 °C for 5 min, 35 cycles of denaturation at 94 °C for 45 s, annealing (Table 1) for 45 s, extension at 72 °C for 45 s, and a final extension at 72 °C for 7 min.

**Table 1:** Primers for PCR amplification of *E. coli* O157:H7

Target gene	Primer sequence (5’−3’)	Product size(bp)	Annealing temperature (°C)	Reference
*O157*	F: CGGACATCCATGTGATATGG R: TTGCCTATGTACAGCTAATCC	259	52	(Tayh et al. 2022)
*fliCH7*	F: GCGCTGTCGAGTTCTATCGAGC R: CAACGGTGACTTTATCGCCATTCC	625	60	
*uidA*	F: ATCACCGTGGTGACGCATGTCGC R: CACCACGATGCCATGTTCATCTGC	486	56	
*Stx1*	F: AGTTAATGTGGTGGCGAAGG R: CACCAGACAATGTAACCGC	347	58	(Altaie et al. 2025)
*Stx2*	F: TTCGGTATCCTATTCCCGG R: CGTCATCGTATACACAGGAG	592	58	
*eaeA*	F: CACACGAATAAACTGACTAAAATG R: AAAAACGCTGACCCGCACCTAAAT	376	55	
*hlyA*	F: ACGATGTGGTTTATTCTGGA R: CTTCACGTGACCATACATAT	167	50	
*tetA*	GGTTCACTCGAACGACGTCA CTGTCCGACAAGTTGCATGA	576	50	(Younis et al. 2021)
*blaTEM*	ATCAGCAATAAACCAGC CCCCGAAGAACGTTTTC	516	54	
*Aada1*	TATCAGAGGTAGTTGGCGTCAT GTTCCATAGCGTTAAGGTTTCATT	484	54	(Omara et al. 2024)

### Virulence and resistant genes in *E. coli* O157:H7

Verified *E. coli* O157:H7 isolates were tested for the detection of various virulence genes, including *Stx1* (encoding Shiga toxin 1), *Stx2* (encoding Shiga toxin 2), *eaeA* (encoding intimin), *uidA* (aerobactin system), and *hlyA* (encoding haemolysin), as well as antibiotic-resistant genes such as *tetA* (tetracyclines), *blaTEM* (beta-lactams), and *Aada1* (aminoglycosides). Table 1 contains all of the primers, annealing temperatures, and the size of the amplified fragments for every gene.

The PCR reaction includes 12.5 µL of COSMO PCR RED Master Mix, 1 µL each of reverse and forward primers (20 pmol), 6 µL of DNA, and 4.5 µL of free nuclease water. The process involved: denaturation at 95 °C for 5 min, 30 cycles of 30 s at 95 °C and 30 s at the annealing temperatures (Table 1), and 72 °C for 30 min, and a final extension at 72 °C for 7 min. A PCR mix without any DNA template served as a negative control. In contrast, locally derived isolates from the Animal Health Research Institute, Egypt, carrying targeted virulence genes served as positive controls. The PCR products were electrophoresed on a 1% agarose gel at 50 volts, stained with ethidium bromide, and subsequently observed under UV light to facilitate the visualisation of band migration using a 100 bp ladder (El-Hawary et al. 2025).

### Antibiotic sensitivity of *E. coli* O157:H7

The Kirby–Bauer disc diffusion technique on Mueller–Hinton agar was employed to assess the in vitro antimicrobial susceptibility of all confirmed *E. coli* O157:H7 isolates towards 15 agents from ten distinct antimicrobial classes, following the National Committee for Clinical Laboratory Standards Institute (CLSI 2021). The antibiotic discs (Shanghai Yuanye Bio-Technology Co., Shanghai, China) were involved aminoglycosides (Gentamycin [GT], 10 µg; kanamycin [KAN], 30 µg), phenicols (chloramphenicol [CPL], 30 µg; florfenicol [FLO], 30 µg), β-lactams (ampicillin [AMP], 10 µg; penicillin G [PEN], 10 µg), fluoroquinolones (Ciprofloxacin [CIP], 5 µg; enrofloxacin [ENR], 10 µg), a lipopeptide (polymyxin B [PB], 300 µg), sulfonamides (sulfamethoxazole-trimethoprim [SLT), 1.25–23.75 µg), tetracycline (TET, 30 µg), macrolide (erythromycin [EM],15 µg; azithromycin [AZM], 15 µg), quinolone (nalidixic acid [NAL], 30 µg), rifamycins (rifampin [RIF],30 µg). These antibiotics were chosen due to their accessibility and repeated use in the research region, both in veterinary and human medicine.

Moving pure colonies grown on nutrient agar (M001, HiMedia) to a 5 mL tryptone soya broth (TSB; M2059, HiMedia) and keeping it at 37 °C for 18 h, the growth was checked until it matched the 0.5 McFarland turbidity standards (Oxoid). The swab was then left at room temperature for 30 min to dry. A sterile cotton swab (PW1317, HiMedia) was dipped in the suspension and thoroughly wiped over the surface of Mueller–Hinton agar plates (PO0152, Oxoid). The swab was then left at room temperature for 30 min to dry. The antibiotic discs were carefully pressed onto the agar surface using sterile forceps to ensure strong contact on the inoculated plates. The plates were kept at 37 °C for 24 h (Mohamed et al. 2025). The level of resistance to the respective antibiotics was detected by calculating the diameters of the inhibition zone after incubation with a caliper. The isolates were categorized as sensitive (S), intermediate (I), and resistant (R) regarding the CLSI (2021). *E. coli* ATCC 25,922 from the Animal Health Research Institute in Egypt was used as a quality control strain. The multiple-antibiotic resistance (MAR) indices were calculated by dividing the number of antibiotics to which the isolate was resistant by the total number of antibiotics analyzed. A MAR rating of < 0.2 pointed to the fact that the isolates came from a low-risk, contaminated source. Conversely, isolates from high-risk pollutants show MAR > 0.2 (Qenawy et al. 2024).

### In vitro assessment of antibacterial activity of PcL extract against *E. coli* O157:H7

#### Collection and processing of the PcL

PcL berries were acquired from the Agricultural Research Center (ARC, Giza, Egypt), brought for analysis at the Food Hygiene Laboratory, Faculty of Veterinary Medicine, Aswan University, and dried at 60 °C in a drying oven (BINDER, ED53, Germany). The dried berries were ground into a fine powder using a strong blender (OEM, J-1700, China). The powdered PcL was kept in a refrigerator at −80 °C in an airtight polyethylene plastic bag (Rahman et al. 2023).

#### PcL extraction

In brief, 200 g of dried PcL berries were ground with a dry blender (OEM, J-1700, China). The ground berries were soaked in a 1:10 ratio of 99.8% ethanol (Sigma-Aldrich, Missouri, USA) for 48 h at room temperature with standard shaking. Plant extract was filtered by Whatman filter paper size No. 2 (Whatman International Ltd., Middlesex, England). Subsequently, the extracts were concentrated at 40 °C for 3–4 h using a rotary vacuum evaporator (SKU: JA21650-68, India) to produce an ethanol extract of the desiccated PcL berries. To ensure that the extract was devoid of methanol, the temperature of the rotary evaporator was elevated to 85 °C twice for 30 s at the end of the extraction process. Lastly, the extracts were freeze-dried for 48 h to remove water and kept at 4 °C until they were used (Alqadeeri et al. 2020).

Ethanolic PcL extract was prepared by diluting 1 mL of extract into 99 mL of 10% dimethylsulfoxide (DMSO; Fisher Scientific, Leicestershire, United Kingdom) to a final concentration of 1% for evaluation of the antibacterial action on *E. coli* O157:H7 using the disc diffusion assay (DDA), minimum inhibitory concentration (MIC), minimum bactericidal concentration (MBC), and time-kill curve assay. Further dilutions of the extracts were prepared at 5%, 3%, 1%, and 0.5% concentrations by adding 5 mL, 3 mL, 1 mL, and 0.5 mL of extract into 95 mL, 97 mL, 99 mL, and 99.5 mL of 10% DMSO, respectively. Sterile deionized water was used as a 0% PcL extract concentration.

#### Disc diffusion assay (DDA)

A pure isolate of *E. coli* O157:H7 was subculture on the nutrient agar (M001, HiMedia) plates and kept at 37˚C for 24 h. A disk diffusion test was done following the procedure of CLSI (2012). Using 0.9% sterile saline solution, the suspension turbidity of purified *E. coli* O157:H7 culture was adjusted equal to that of 10^6^ cfu/mL (standardized by 0.5 MCFarland standard). Subsequently, a sterile cotton swab was employed for spreading 100 µL of the sample onto Mueller-Hinton agar (PO0152, Oxoid) plates. Using a sterilized cork borer, holes (7 mm) were created in the plates. These were then filled with 100 µL of produced PcL extract of 3%, 1%, and 0.5% and incubated at 37 °C for 24 h. A negative control was recognized by sterile demineralized water, although a positive control was established via antibiotic discs (ampicillin, 10 µg). The inhibitory halo diameter was measured using a gauge (mm). Assessments were taken in triplicate to establish the mean and standard deviations of the inhibition zone, which were determined. The strains were classified as resistant (0) for diameters ˂ 8 mm, moderately sensitive (+) for 8–20 mm, sensitive (++) for 20–30 mm, and very sensitive (+++) for diameters ˃ 30 mm.

#### Assessment of minimum inhibitory concentration (MIC) and minimum bactericidal concentration (MBC)

Using the guidelines of CLSI (2012), the MIC and MBC were measured using a standard broth microdilution method. Fresh Mueller-Hinton broth (MHB; CM405B, Oxoid) was used to create the bacterial suspensions for the experiment, and the bacteria concentration was set to match 10^6^−10^8^ CFU/mL (standardized by the 0.5 McFarland standard). 100 µL MHB was served as a negative control in Column 1, and 100 µL bacterial suspension with medium served as a positive control in Column 2. The microdilution was done at extract concentrations from 5.000 mg/mL to 0.009 mg/Ml (CLSI 2012). 100 µL of each PcL dilution was poured on U-shaped bottom, sterile polystyrene, 96-well culture plates (Techno Plastic Products, Switzerland). Every well got 100 µL of every bacterial suspension, kept at 37 °C for 24 h. The MBC was recognized by subculturing 10 µL of the suspension from every well on MHA. The plates were subsequently left at 37 °C for 24 h, or until growth was detected in the positive growth control. The MIC was found to be the smallest amount of antibacterial agent that completely stopped visual growth (CLSI 2012). This means that there was no growth in the well that was related to the positive and negative growth wells. The MBC was recognized as the lowest dose that produced no observable growth following the incubation period (Andrews 2001). Every test was approved in triplicate, and the mean ± standard error of the mean was used to present the findings.

#### Time-kill curve studies

The ability of PcL to kill the *E. coli* O157:H7 strain being studied was measured by using a time-kill curve following the (CLSI 2012). This methodology enables the assessment of the bactericidal efficacy of PcL and the acquisition of information regarding the dynamics of this action. MHB holding roughly 10^6^ CFU/mL of inoculum suspension was mixed with the extract at 0 × MIC, 1 × MIC, 2 × MIC, and 3 × MIC until the final volume was 1 mL. After that, the mixture was incubated at 37 °C while being stirred at 200 rpm. The microdilution method was used to check the viability of *E. coli* O157:H7 after PcL treatment at different incubation times (0, 1, 4, 8, and 12 h). Using the spread plate technique, 1 mL of every sample was serially diluted and plated on tryptic soy agar. After 24 h of incubation at 37 °C, colonies were enumerated. The total plate count (TPC) was determined, and the time-kill curve was constructed by plotting the log_10_ of bacteria versus time (Gomez-Llorente et al., 2025). Three separate analyses of each treatment were performed.

#### Statistical analysis

GraphPad Prism 8 (GraphPad Software Inc.; San Diego, CA, USA) was employed for data analysis, applying a one-way ANOVA test. The chi-square test was used to determine the relationship between category variables. The MIC and MBC data were analyzed using a two-sample T-test, revealing a significant difference (*p* < 0.01) between the treatment and positive control. The antimicrobial profile of *E. coli* O157:H7 isolates from various sources to various tested antimicrobial agents was visualized using online software using a heatmap (heatmap v7.0.1) and hierarchical bars (Hierarchy v24.1.1). A probability (*p*) value of < 0.05 was regarded statistically significant.

## Results

### Occurrence of *E. coli* and *E. coli *O157:H7

The initial diagnosis was based on the appearance and chemical tests of the apparent *E. coli* colonies, which are pink-colored with a rod shape on MacConkey agar and dark-centered and flat colonies with a metallic sheen on EMB agar. The probable *E. coli* colonies were Gram-negative, and by biochemical tests indicated that the colonies were positive for methyl red, L-lysine decarboxylation, lactose, and indole but negative for oxidase, citrate utilization, urease, VP, and H2S production. Additionally, the presumptive *E. coli* O157:H7 colonies appeared as sorbitol non-fermenting (colorless or pale) on sorbitol MacConkey agar.

Out of 410 examined samples from food products, animals, water, and human stool, 115 (28%) have *E. coli*, with an *E. coli* O157:H7 incidence of 8%, as shown in Table 2. The outcomes revealed that *E. coli* was found in 24.2% of food products, and 7.5% of those were *E. coli* O157:H7 (*p* ˃ 0.05); 25% of animal feces had *E. coli*, and 6% were *E. coli* O157:H7 (*p* ˃ 0.05); and 26.7% of water samples contained *E. coli*, with 8.3% being *E. coli* O157:H7 (*p* ˃ 0.05). Furthermore, the comparisons revealed that the occurrence of *E. coli* and *E. coli* O157:H7 in human stool samples was much higher (*p* < 0.05) than in other sources, with rates of 34.6% for *E. coli* and 10.8% for *E. coli* O157:H7.

**Table 2:** Occurrence of *E. coli* and *E. coli* O157:H7 across various sources

Samples source		No. of samples	Positive samples		Other E. coli serotype		E. coli O157: H7	
			No.	%	No.	%	No.	%
Food product	Minced beef	20	5	25.0	4	22.0	1	5.0
	Beef kofta	20	6	30.0	4	22.0	2	10.0
	Beef burger	20	6	30.0	4	22.0	2	10.0
	Raw cow milk	20	5	25.0	3	15.0	2	10.0
	Karish cheese	20	4	22.0	3	15.0	1	5.0
	Yoghurt	20	3	15.0	2	10.0	1	5.0
	Sub-total N (%)	120	29	24.2	20	16.7	9	7.5
	Chi^2^		0.532		0.346		0.742	
	*p*-value		0.979		0.990		0.9511	
Animal fecal	Ox	30	7	23.3	6	20.0	1	3.3
	Cow	30	10	33.3	7	23.3	3	10
	Sheep	20	4	20.0	3	15.0	1	5.0
	Goat	20	4	20.0	3	15.0	1	5.0
	Sub-total N (%)	100	25	25.0	19	19.0	6	6.0
	Chi^2^		0.985		0.547		1.134	
	*p*-value		0.805		0.908		0.768	
Water	Nasser Lake	30	7	23.3	5	16.7	2	6.7
	River Nile	30	9	30.0	6	20.0	3	10.0
	Sub-total N (%)	60	16	26.7	11	18.3	5	8.3
	Chi^2^		0.196		0.078		0.185	
	*p*-value		0.657		0.782		0.667	
Human stool	Diarrheic	90	38	42.2	28	30.0	10	11.1
	Apparently healthy	40	7	17.5	4	10.0	3	7.5
	Sub-total N (%)	130	45	34.6	32	23.8	13	10.8
	Chi^2^		3.939		4.332		0.333	
	*p*-value		0.047		0.037		0.564	
Total No. (%)		410	115	28.0	82	20.0	33	8.0

### Incidence of *E.coli* and *E. coli* O157:H7 in human samples

The incidence of *E. coli* and *E. coli* O157:H7 in humans in terms of gender, age, and residence is shown in Table 3. Furthermore, the variation between each variable was not statistically significant (*p* ˃ 0.05). The outcomes of the statistical study disclosed that *E. coli* and *E. coli* O157:H7 was associated with age as a risk factor; among the individuals with diarrhea, the highest incidence was in the group of ˂ 15 years, followed by the age group ˃ 41, while among the 16–41 years group. In comparison with the healthy, the highest incidence was in the group of > 41 years, followed by the 16–41 years group for each *E. coli* and *E. coli* O157:H7, while among the age group ˂ 15. In addition, the incidence of *E. coli* and *E. coli* O157:H7 among the individuals with diarrhea was more frequent in those residing in rural than in areas. Furthermore, the incidence among apparently healthy individuals was 13% and 8.7% in rural and 5.9% in urban areas.

**Table 3:** Frequency distribution of *E. coli* and *E. coli* O157:H7 in humans in relation to gender, age, and residence

Variable		No.	Diarrheic (*n* = 38)		No.	Apparently healthy (*n* = 7)	
			Other *E. coli* serotype no. (%)	*E. coli* O157:H7 no. (%)		Other *E. coli* serotype no. (%)	*E. coli* O157:H7 no. (%)
Gender	Male	45	19 (42.2)	7 (15.6)	20	2 (10.0)	2 (10.0)
	Female	45	9 (20.0)	3 (6.7)	20	2 (10.0)	1 (5.0)
	Chi^2^			0.016			0.194
	*p*-value			0.900			0.659
Age (year)	˂15	41	14 (34.1)	6 (14.6)	22	2 (9.1)	1 (4.5)
	16–40	33	9 (27.3)	2 (6.1)	10	1 (10.0)	1 (10.0)
	˃41	16	5 (31.3)	2 (12.5)	8	1 (12.5)	1 (12.5)
	Chi^2^			0.533			0.194
	*p*-value			0.765			0.907
Residence	Urban	37	11 (30.0)	3 (8.1)	17	1 (5.9)	1 (5.9)
	Rural	53	17 (32.1)	7 (13.2)	23	3 (13.0)	2 (8.7)
	Chi^2^	0.273					0.058
	*p*-value	0.601					0.809

### Serotyping of *E. coli* isolates

**Fig. 1:**
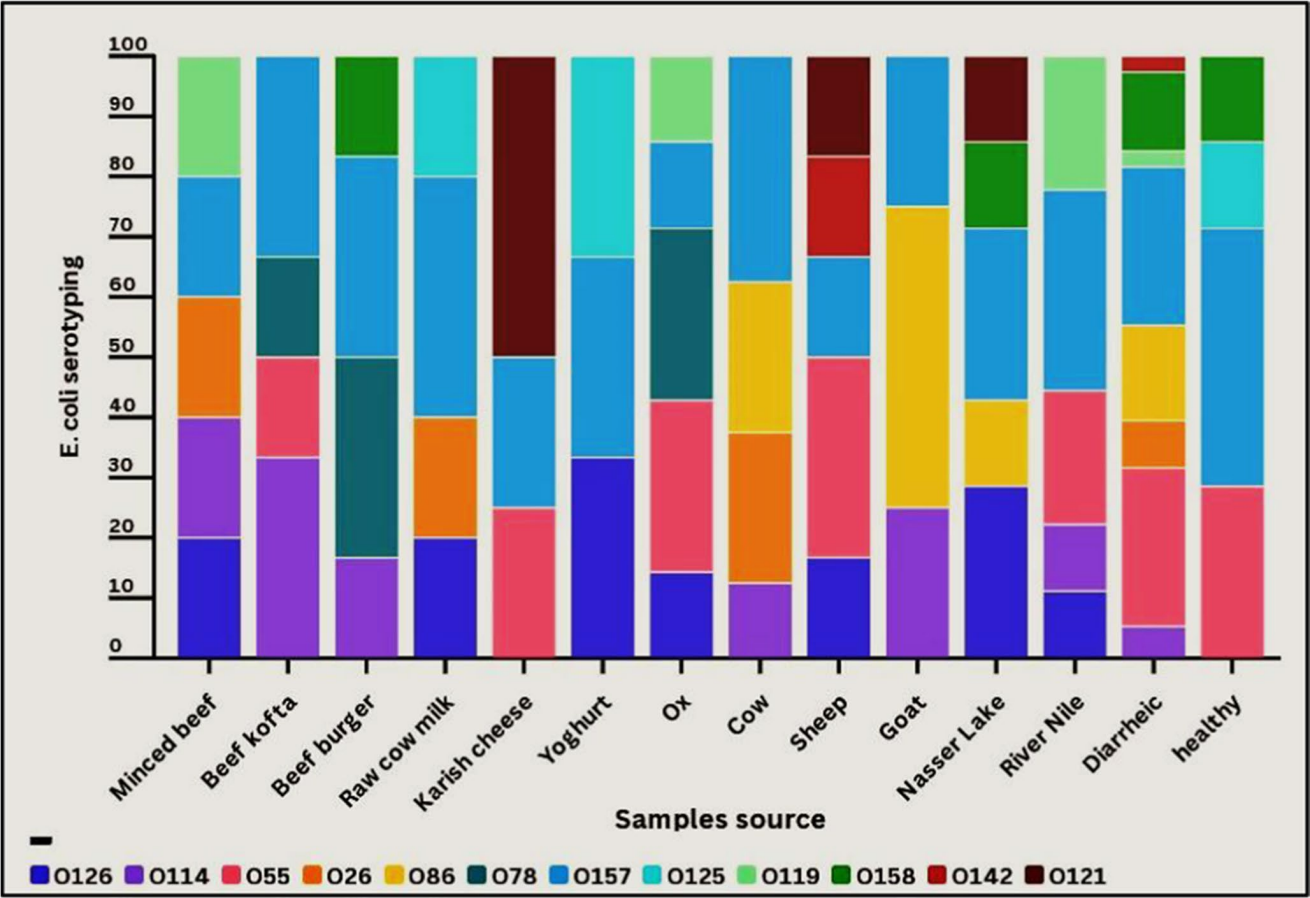
Serotyping of *E. coli* strains isolated from different sources

The serological analysis using the latex agglutination test revealed the detection of 12 distinct *E. coli* serotypes (Fig. 1 and Supplementary Table S1). The most predominant serotypes identified were O157 (28.7%), O55 (17.4%), O86 (9.6%), O114 (7.8%), O126 (7%), O158 (7%), O26 (5.2%), O78 (4.3%), O119 (4.3%), O121 (3.4%), O125 (2.6%), and O142 (1.7%).

### Genome profile of E. coli O157:H7 by multiplex PCR

**Fig. 2:**
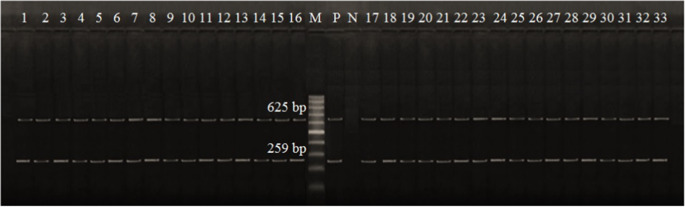
Electrophoretic profile of amplification products of the *O157* gene at 259 bp and *fliCH7* gene at 625 bp in *E. coli* O157: H7 isolated from the examined samples. Lane 1: isolate from minced beef, Lanes 2–3: isolates from beef kofta, Lanes 4–5: isolates from beef burger, Lanes 6–7: isolates from raw cow milk, Lane 8: isolate from karish cheese, Lane 9: isolate from yoghurt, Lane 10: isolate from ox fecal matter; Lanes 11–13: isolates from cow fecal matter; Lane 14: isolate from sheep fecal matter; Lane 15: isolate from goat fecal matter; Lanes 16–17: isolates from Nasser Lake water, Lanes 18–20: isolates from River Nile water; Lanes 21–30: isolates from diarrheic individuals; and Lanes 31–33: isolates from apparently healthy individuals. M: marker. P: positive control. N: negative control

**Fig. 3:**
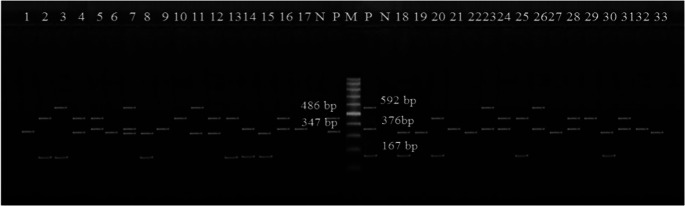
Electrophoretic profile of amplification products of virulence genes in *E. coli* O157: H7: *uidA* at 486 bp, *Stx1* at 347 bp, *Stx2* at 592 bp, *eaeA* at 376 bp, and *hlyA* at 167 bp. Lane 1: isolate from minced beef, Lanes 2–3: isolates from beef kofta, Lanes 4–5: isolates from beef burger, Lanes 6–7: isolates from raw cow milk, Lane 8: isolate from karish cheese, Lane 9: isolate from yoghurt, Lane 10: isolate from ox fecal matter; Lanes 11–13: isolates from cow fecal matter; Lane 14: isolate from sheep fecal matter; Lane 15: isolate from goat fecal matter; Lanes 16–17: isolates from Nasser Lake water, Lanes 18–20: isolates from River Nile water; Lanes 21–30: isolates from diarrheic individuals; and Lanes 31–33: isolates from apparently healthy individuals. M: marker. P: positive control. N: negative control

**Fig. 4:**
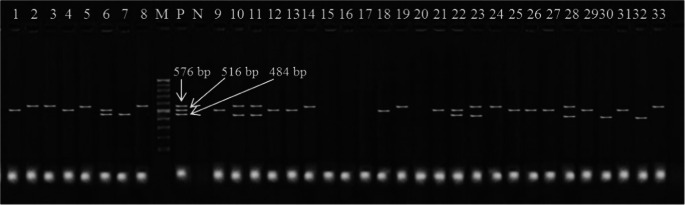
Electrophoretic profile of amplification products of antibiotic-resistant genes in *E. coli* O157: H7: *tetA* at 576 bp, *blaTEM* at 516 bp, and *Aada1* at 484 bp. Lane 1: isolate from minced beef, Lanes 2–3: isolates from beef kofta, Lanes 4–5: isolates from beef burger, Lanes 6–7: isolates from raw cow milk, Lane 8: isolate from karish cheese, Lane 9: isolate from yoghurt, Lane 10: isolate from ox fecal matter; Lanes 11–13: isolates from cow fecal matter; Lane 14: isolate from sheep fecal matter; Lane 15: isolate from goat fecal matter; Lanes 16–17: isolates from Nasser Lake water, Lanes 18–20: isolates from River Nile water; Lanes 21–30: isolates from diarrheic individuals; and Lanes 31–33: isolates from apparently healthy individuals. M: marker. P: positive control. N: negative control

**Fig. 5:**
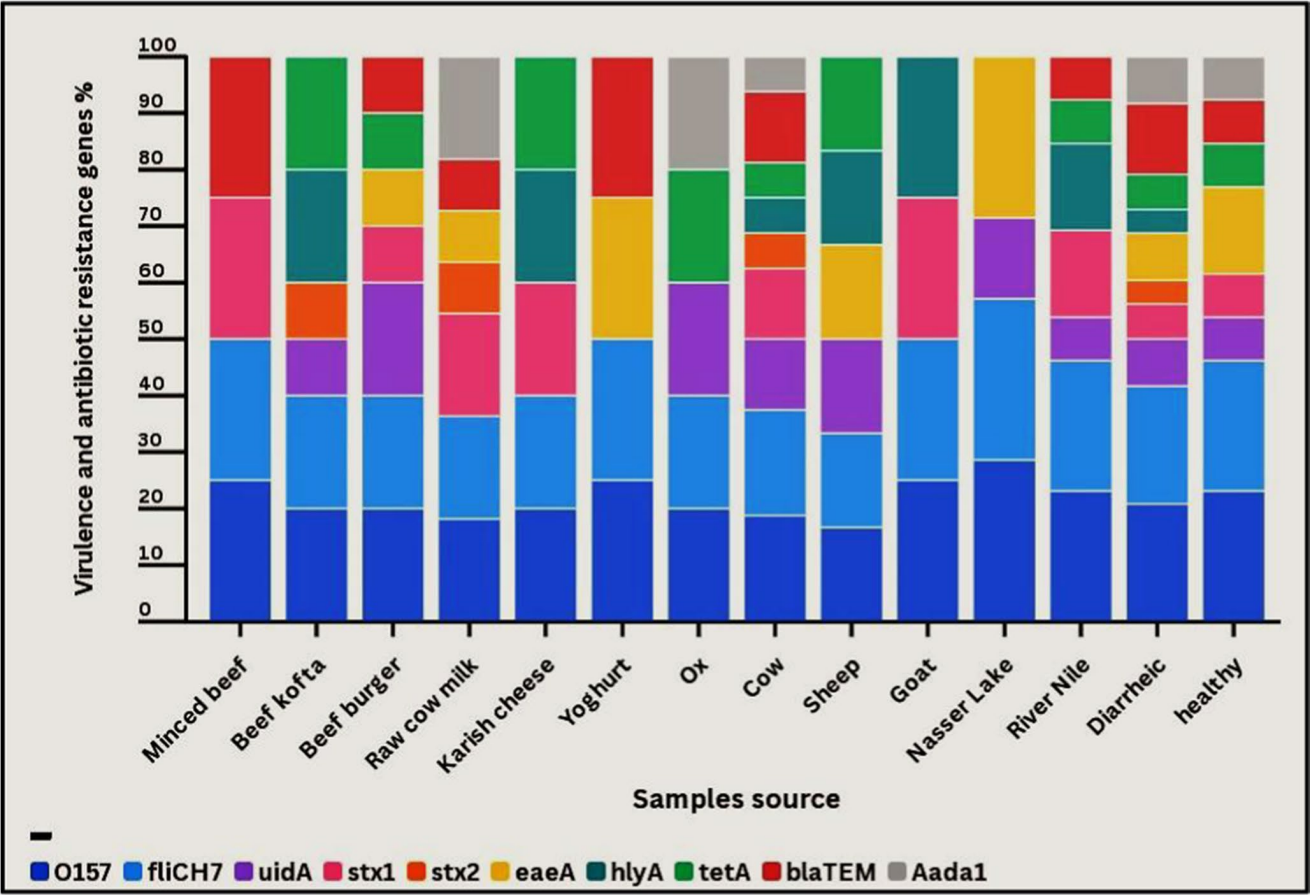
Distribution of conformation genes (*O157* and *fliCH7*), virulence (*uidA*, *Stx1*, *Stx2*, *eaeA*, and *hlyA*), and antibiotic resistance genes (*tetA*, *blaTEM*, and *Aada1*) in *E. coli* O157:H7 strains that were isolated from the examined samples

The PCR experiment was conducted on serologically classified *E. coli* O157:H7 strains via primers specified for virulence and antibiotic resistance genes. All of these isolates were verified as *E. coli* O157:H7 through detection of the *rfbO157* and *flicH7* genes using appropriate primers (Fig. 2). PCR examination of the 33 *E. coli* O157:H7 isolates confirmed that the *uidA*, *Stx1*, *Stx2*, *eaeA*, and *hlyA* virulence genes (Fig. 3) occurred in 39.4%, 42.4%, 15.2%, 36.4%, and 30.3%. In contrast, *tetA*, *blaTEM*, and *Aada1* antibiotic resistance genes (Fig. 4) were present in 36.4%, 42.4%, and 27.3%. The current finding showed that the rates of virulence and antibiotic resistance genes vary along with the source of the sample, despite belonging to a similar serotype, indicating a statistical difference (*p* < 0.05) (Fig. 5 and Supplementary Table S2).

### Antibiogram profile of E. coli O157:H7

**Fig. 6:**
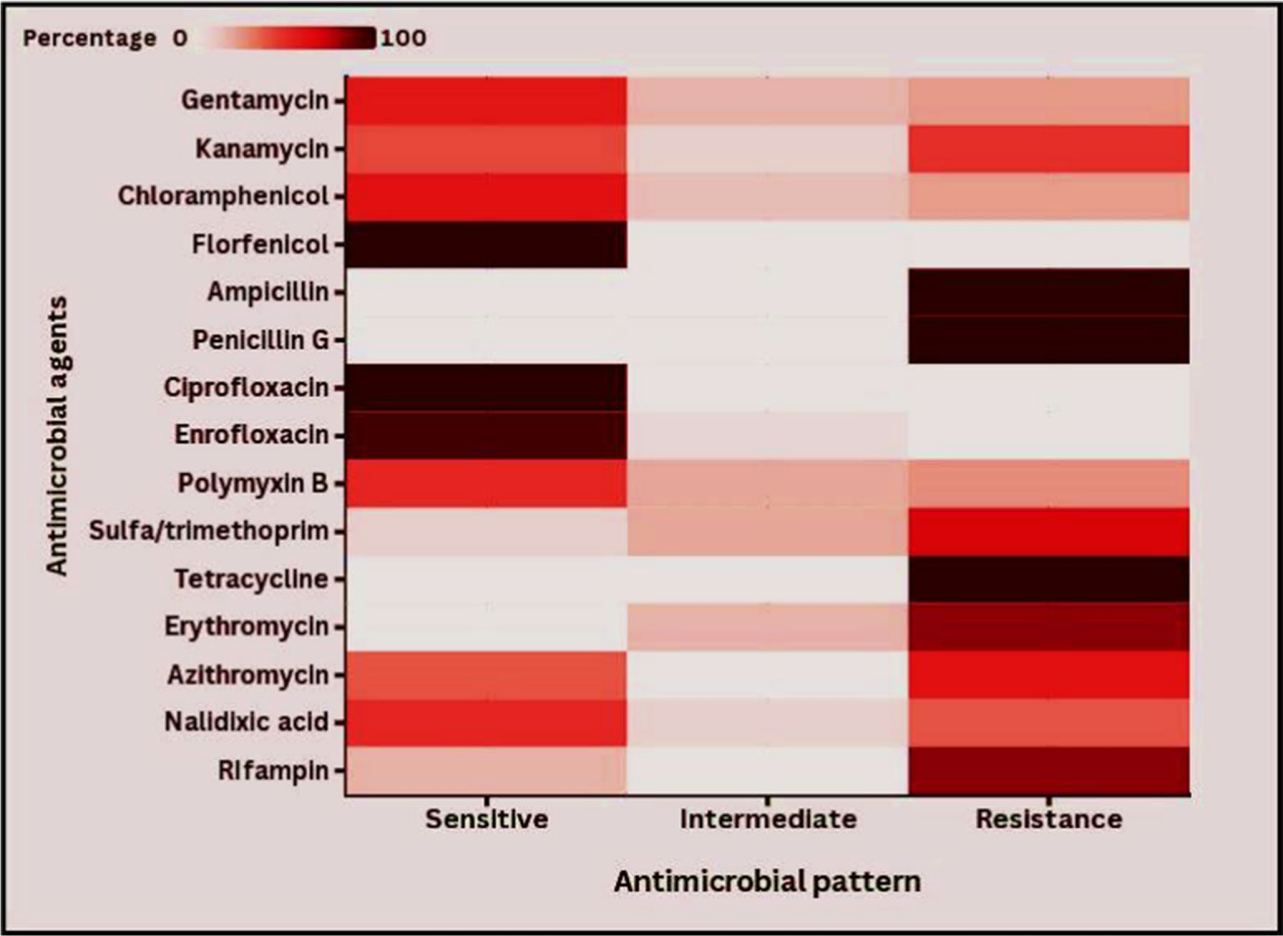
Heatmap showing the antimicrobial profile of *E. coli* O157:H7 isolates to various tested antimicrobial agents, where darker colors represent a higher susceptibility. *p* < 0.0001

**Fig. 7:**
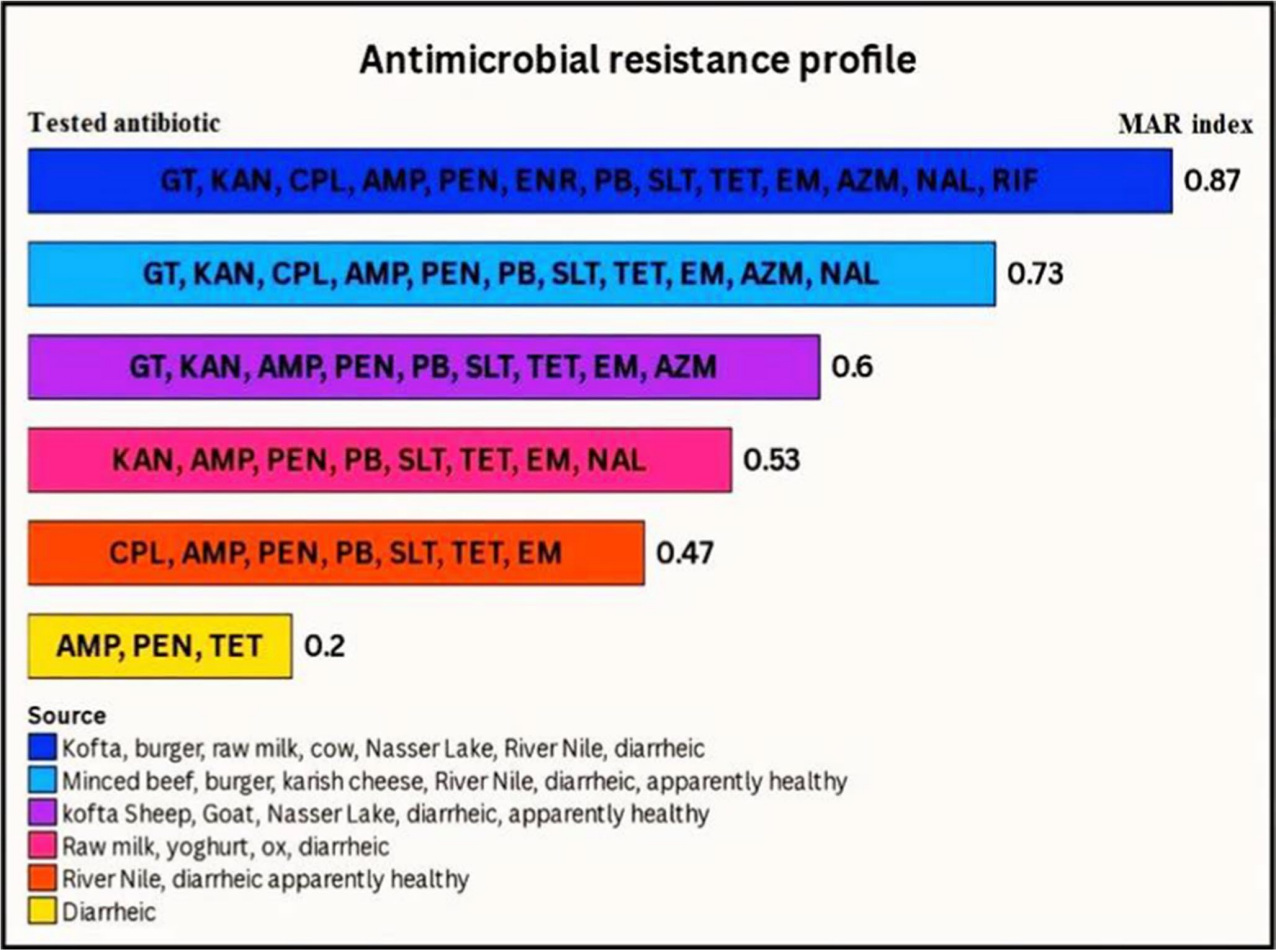
Hierarchical analysis for the determination of antibacterial resistant profile of *E. coli* O157:H7 isolates (*n* = 33). MAR: multiple antibiotics resistant. Gentamycin (GT), kanamycin (KAN), chloramphenicol (CPL), florfenicol (FLO), ampicillin (AMP), penicillin G (PEN), Ciprofloxacin (CIP), enrofloxacin (ENR), polymyxin B (PB), sulfamethoxazole-trimethoprim (SLT), tetracycline (TET), erythromycin (EM), azithromycin (AZM), nalidixic acid (NAL), rifampin (RIF)

The current study of 33 *E. coli* O157:H7 samples tested 15 different antimicrobial agents and found significant patterns of resistance and sensitivity (*p* < 0.05), which are important for public health (Fig. 6 and Supplementary Table S3). Most strains were resistant to AMP, PEN, and TET (100% each). The tests indicated that the bacteria under study were highly resistant to EM, SLT, AZM, KAN, NAL, PB, GT, CPL, and RIF, with resistance rates of 81.1%, 69.7%, 60.6%, 48.5%, 39.4%, 27.3%, 24.2%, 24.2%, and 18.2%, respectively. Additionally, the tested isolates showed some resistance to PB (21.2%), SLT (21.2%), GT (18.2%), EM (18.2%), CPL (15.2%), KAN (9.1%), NAL (9.1%), and ENR (6%). Furthermore, the most significant antibiotics were FLO and CIP, which showed complete sensitivity (100% each), followed by ENR (94%), RIF (81.8%), and CPL (60.6%). The results also indicate that the tested isolates were responsive to GT (57.6%), NAL, and PB (51.5% each), KAN (42.2%), AZM (39.4%), and SLT (9.1%). The *E. coli* O157:H7 strain was resistant to multiple drugs (at least three types of antibiotics) with levels ranging from 0.200 to 0.867 and an average of 0.567 (Fig. 7 and Supplementary Table S4).

### Antimicrobial effect of PcL on E. coli O157:H7

The agar diffusion test showed that all doses of PcL (0.5%, 1%, 3%, and 5%) significantly reduced the development of 15 *E. coli* O157:H7 strains (*p* < 0.05), with higher amounts of the extract leading to larger areas where bacteria were inhibited. The inhibitory zone diameter was between 5.2 ± 0.7 mm and 7.4 ± 0.6 mm (0.5% PcL), 6.8 ± 0.4 mm and 9.7 ± 0.4 mm (1% PcL), 9.2 ± 0.6 mm and 14.2 ± 0.8 mm (3% PcL), and 11.4 ± 0.7 mm and 17.7 ± 0.5 mm (5% PcL). MIC and MBC were conducted using 15 *E. coli* O157:H7 isolates to determine the precise antibacterial qualities of PcL. The MIC data revealed that PcL had high antibacterial action, contrary to the tested isolates, with MICs varying from 0.43 to 0.74 mg/ml. In the case of PcL being a bactericidal agent, some of the tested isolates showed an MBC rate that was about the same as the MIC rate (Table 4).

**Table 4:** Inhibitory zone diameters, MIC, and MBC of *PcL* against *E. coli O157* isolates (*n* = 15)

Isolate no.	Zone diameter (mm) against various PcL concentrations (%)				MIC (mg/mL)	MBC (mg/mL)
	0.5	1	3	5		
1	5.3 ± 0.5	8.0 ± 0.4	10.0 ± 0.4	13.0 ± 0.7	0.43	0.86
2	6.5 ± 0.4	7.6 ± 0.2	9.2 ± 0.6	11.4 ± 0.7	0.43	0.43
3	6.0 ± 0.7	9.2 ± 0.7	11.4 ± 0.5	14.2 ± 0.2	0.67	1.34
4	5.7 ± 0.4	7.3 ± 0.5	9.8 ± 0.4	12.5 ± 0.1	0.44	0.88
5	7.2 ± 0.3	8.8 ± 0.2	11.3 ± 0.7	14.6 ± 0.3	0.43	0.86
6	5.5 ± 0.3	7.2 ± 0.7	9.5 ± 0.3	13.6 ± 0.2	0.65	1.30
7	5.2 ± 0.7	6.8 ± 0.4	10.2 ± 0.2	13.9 ± 0.6	0.67	0.67
8	6.7 ± 0.4	9.2 ± 0.5	12.7 ± 0.4	17.4 ± 0.3	0.74	1.48
9	7.4 ± 0.6	9.3 ± 0.7	11.8 ± 0.5	14.6 ± 0.7	0.43	0.86
10	6.6 ± 0.3	8.6 ± 0.1	12.2 ± 0.6	15.4 ± 0.3	0.65	1.30
11	7.1 ± 0.6	9.7 ± 0.3	13.5 ± 0.3	17.2 ± 0.4	0.74	0.74
12	5.8 ± 0.6	9.2 ± 0.5	13.3 ± 0.6	16.1 ± 0.5	0.74	1.48
13	6.5 ± 0.4	7.6 ± 0.2	9.2 ± 0.6	11.4 ± 0.7	0.67	0.67
14	7.3 ± 0.7	9.7 ± 0.4	14.2 ± 0.8	17.7 ± 0.5	0.44	1.76
15	5.7 ± 0.2	7.6 ± 0.4	10.2 ± 0.5	14.8 ± 0.8	0.67	1.34

*PcL*: *Piper cubeba L*. MIC: Minimal inhibitory concentration. MBC: minimal bactericidal concentration. *p* value was significant different at *p* < 0.05

### Time-kill curve

**Fig. 8:**
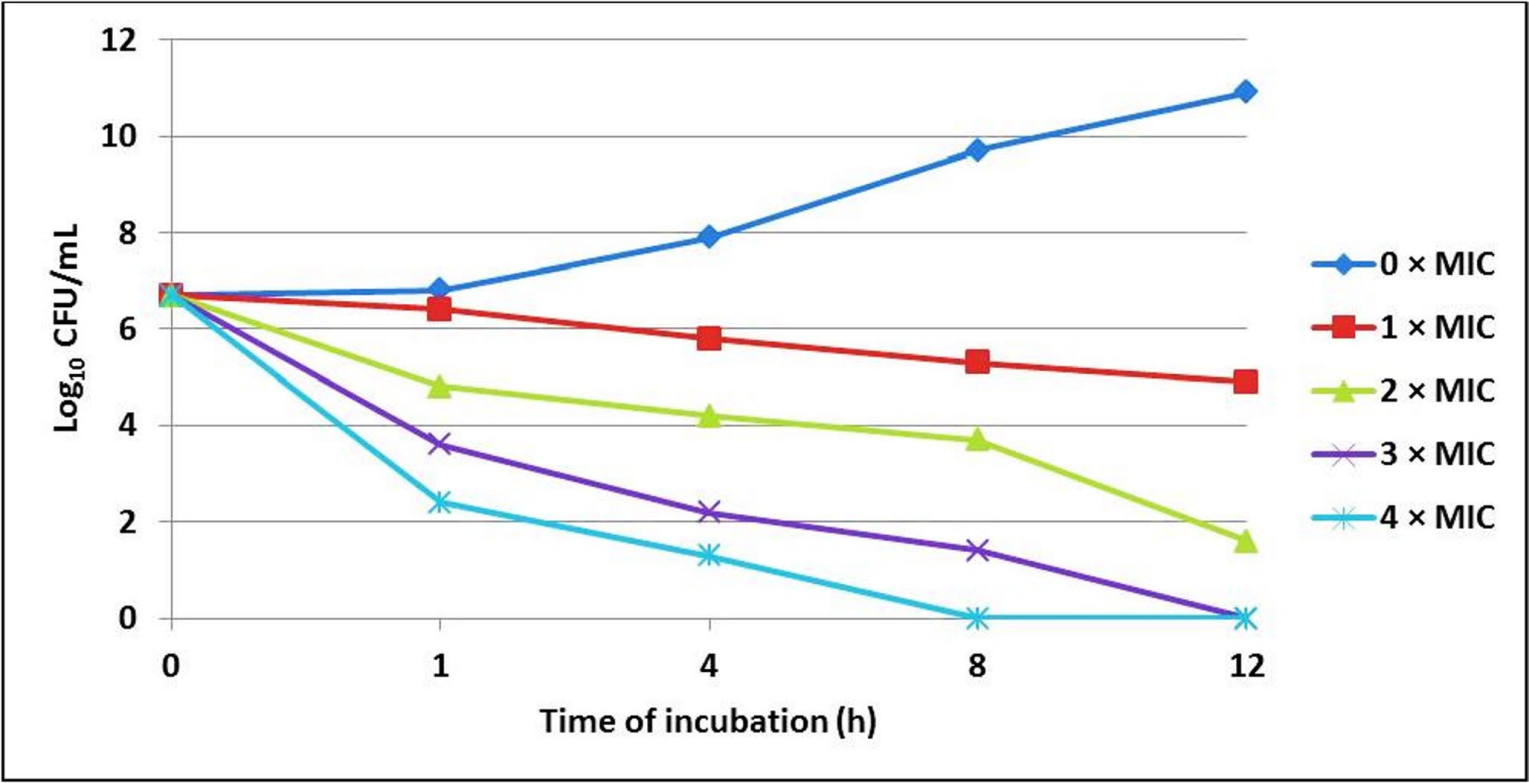
Time-kill curve plot of *E. coli* O157:H7 after different exposure times to different *Piper cubeba Linn* concentrations

Figure 8 and Supplementary Table S5 plot the time-kill curve of the PcL extract against *E. coli* O157:H7. The curve shows that the number of bacteria in the sample without PcL extract (0.0 × MIC) increased from 6.7 to 10.9 log_10_ CFU/mL over 12 h. The samples treated with 1 × MIC (0.43 mg/mL) of PcL extract showed a slight decrease in bacteria over 12 h. The bacterial count dropped significantly (*p* < 0.05) in the samples treated with 2 × MIC (0.86 mg/mL), 3 × MIC (1.29 mg/mL), and 4 × MIC (1.72 mg/mL). The results indicated that the PcL extract was effective at killing *E. coli* O157:H7 at a concentration of 1.29 mg/mL after 12 h and at 1.72 mg/mL after 8 h.

## Discussion

*E. coli*, particularly *E. coli* O157:H7, a serious bacterium that can cause food poisoning, poses a considerable threat to food safety and public health worldwide by spreading from animals to humans via the consumption of undercooked meat, drinking unpasteurized milk, using polluted water, or interacting with animal waste (Dandrawy et al. 2025). In the current study, 28% of the studied specimens had *E. coli*, with an *E. coli* O157:H7 incidence of 8%, and the rates of *E. coli* and *E. coli* O157:H7 in human stool specimens were much higher (*p* < 0.05) than in other sources.

The results of *E. coli* and *E. coli* O157:H7 found in humans, animals, and animal products in this study are similar to earlier research in the Middle East and North Africa, where the rates differ a lot based on the country and kind of sample (Altaie et al. 2025; Farag et al. 2023; Jaradat et al. 2024). In comparison to our results, Adzitey et al. (2020) in Ghana, González Gutiérrez (2019) in Spain, and Rajaei et al. (2021) in Iran reported much higher *E. coli* rates of 88%, 100%, and 48%, respectively. In contrast, Abdel-Atty et al. (2023) did not identify *E. coli* in any of Egypt’s analyzed meat product samples. Several studies showed lower rates compared to our research, which found *E. coli* O157:H7 at rates of 25%, 30%, and 30% in minced beef, beef kofta, and beef burger. For instance, a study conducted in Egypt found an incidence of 2.5% (Karmi 2019). Furthermore, the rate in Iraq was lower than ours, with reports of 7.9% (Altaie et al. 2025), while Dandrawy et al. (2025) found much higher rates of 80%, 62.5%, and 67.5% in minced beef, beef kofta, and beef burger, respectively. The variance in detection rates across investigations could be attributed to different samples, natural sources, handling procedures, processing procedures, post-processing contamination, storage conditions, and the use of different laboratory methodologies (Mohammed et al. 2025).

Traditional manual techniques of slaughtering and evisceration in Egypt are frequently carried out in unsanitary conditions, which raise the possibility that ready-to-eat meat products could become contaminated by gut bacteria. According to earlier studies, slaughterhouses lacking automation and with inadequate infrastructure are more vulnerable to microbial contamination because of a lack of sanitary regulations and hygiene procedures (Dandrawy et al. 2025). *E. coli* contamination in milk and dairy products in Egypt is a prevalent issue, suggesting fecal contamination and a potential hazard, as documented by previous studies conducted in various regions and at different times (Elbastawesy et al. 2025; Farag et al. 2023; Ombarak et al. 2016; Younis et al. 2021). The Egyptian Organization for Standardization and Quality (EOS 2020) follows the international standards set by FAO and WHO. These standards include important rules for dairy hygiene, such as keeping the milk at the right temperature, keeping the area clean, ensuring sufficient heat treatment, and avoiding contamination through processing (CAC 2004). However, small farms and street vendors don’t always obey these rules. This emphasizes the significance of closely observing milk production at the small farm and factory level.

Raw milk, karish cheese, and yoghurt in the present study had *E. coli* strains with prevalences of 25%, 22%, and 15%, respectively. This rate is similar to the findings in Egypt, where 20% of raw milk and 16% of karish cheese (Farag et al. 2023), as well as 26.2% of other dairy products (Elbastawesy et al. 2025), were found to have *E. coli*. The current results are lower than the 30.16% of *E. coli* found in raw milk and dairy products in research by Ranjbar et al. (2018) in Iran and also lower than the 77% and 63.3% found by Ombarak et al. (2016) and Taha et al. (2019) in Egypt for different dairy products. In contrast, Omara et al. (2024) and Younis et al. (2021) found that 10% and 19% of raw milk had *E. coli*. In this investigation, potentially pathogenic *E. coli* O157:H7 strains were identified in 10%, 5%, and 5% of raw milk, karish cheese, and yoghurt samples, respectively. This percentage matched with the outcomes from Iraq and Saudi Arabia, where occurrence of 8% and 4.81% was reported (Altaie et al. 2025; Al-Zogibi et al. 2015). Other studies in Egypt found *E. coli* O157:H7 levels from 0% to 6.9% (Jaradat et al. 2024), while Gökmen et al. (2024) found no *E. coli* O157:H7 in raw milk samples in Turkey, suggesting that some places might have much higher levels of contamination. Karish cheese, one of the most popular soft cheeses and inventive dairy products produced in Egypt, is made from fresh unpasteurized milk and most likely under unsanitary conditions before being marketed in local markets, where it may be contaminated with a range of illnesses. The higher occurrence of *E. coli* O157:H7 in karish cheese than in yoghurt could be attributed to the cheese-making procedure and the features of the finished product (Farag et al. 2023).

Cow’s feces samples in the current study showed the highest rates of *E. coli* and *E. coli* O157:H7 compared to ox, sheep, and goats, which matches earlier research on STEC in farm animals (Altaie et al. 2025; Mesele et al. 2023). The concentrated agricultural activities and the normal gut flora of cows are responsible for the high incidence of *E. coli* in comparison to other livestock. *E. coli* O157:H7 was found in cattle feces at an incidence of 4.7% and 4.2%, respectively (Atnafie et al. 2017; Tayh et al. 2022). *E. coli* O157:H7 isolation from feces is thought to be essential for epidemiological understanding, as it can live and proliferate in animal feces for a period exceeding 20 months. Consequently, *E. coli* O157:H7 has the potential to survive in the surroundings and be spread to humans via the ingestion of products that are polluted with animal waste (Dandrawy et al. 2025).

The River Nile and Lake Nasser, which are the main sources of fresh water in Egypt, are important to Egyptians; thus, numerous government and scientific groups work to safeguard them from contamination. The purity of the water is contingent upon the level of industrialization, accessibility of sanitary facilities, the density of the population, and economic conditions. Domestic, agricultural, and industrial wastewaters are the major sources of pollution, along with other human activities such as mining, fishing, oil from ships, etc. (Sayeda 2024). The overall occurrence of *E. coli* and *E. coli* O157:H7 in water samples was 26.7% and 8.3%, with higher rates in the River Nile (30% and 10%) compared to Lake Nasser (23.3% and 6.7%). Still, the variance was not statistically significant (*p* ˃ 0.05), which matches the previous results (Ali et al. 2021; Sayeda 2024). This variation may be because human activity has a lesser impact on Lake Nasser compared to the River Nile. The Nile flows for approximately 1600 km in Egypt, and its waterway takes waste releases from 264 distinct sources, including 121 agricultural drains, 70 industrial outlets, and 73 sewage ejections. Aswan utilizes the EL-Sail Drain to dispose of both domestic and agricultural effluent, along with either treated or untreated sewage. In addition, the water quality is significantly impacted by the industrial wastewater sewage of the Kima factory in El-Sail, Aswan, which discharges approximately 56–70 km³/day of its contaminated water directly into the Nile (Omar et al. 2025).

Our investigation found *E. coli* and *E. coli* O157:H7 in both diarrheal and healthy people, with rates of 34.6% and 10.8%, respectively. This conclusion is consistent with the results of Nji et al. (2021), who demonstrated that *E. coli* O157:H7 was detected in both symptomatic and asymptomatic individuals. These findings have been confirmed by additional research into the incidence of *E. coli* O157:H7 across various clinical conditions. The high levels of *E. coli* found in human stool samples in this study are similar to earlier studies that showed *E. coli* rates in human feces ranging from 20.4% to 55% in different groups (Altaie et al. 2025; Robert et al. 2021), while the rate of *E. coli* O157:H7 in human samples, especially from patients with diarrhea, matched the findings of Abdel-Aziz et al. (2024), who registered a rate of 11%. The investigated diarrheic males (15.6%) and females (6.7%) were sick with *E. coli* O157:H7, which was higher than that in healthy males (10%) and females (5%) without a statistically significant difference. This outcome is much higher than the occurrence rate recorded in various investigations, such as in Iran, which revealed an incidence of 2% (Bonyadian et al. 2024), while in Italy, Muloi et al. (2022) discovered no cases among the examined diarrheal samples. A recent study reported a high prevalence of 62.8% and 45% among diarrheic males and females (Altaie et al. 2025), as well as 50% and 46.4% in another study (Farag et al. 2023). The reason for this finding may be related to behaviour; men tend to travel more frequently than women, which increase their likelihood of consuming raw and undercooked food due to the nature of their jobs as milk distributors, butchers, and slaughterers. Sex factors do not play a role in the biological confirmation of this conclusion (Muloi et al. 2022).

The high incidence of *E. coli* O157:H7 was noted in diarrheal groups < 15 years and ˃ 41 years without a statistically significant dissimilarity (*p* ˃ 0.05). Matched with our findings, Altaie et al. (2025) and Shine et al. (2020) indicated that most of the *E. coli* O157:H7 infections were within the age group < 15 years. One of the causes for the increase in infection prevalence throughout age could be the introduction of supplemental foods and improper food preparation. The ultimate incidence of infection rises as people age, and exposure to different infection sources also varies (Elbastawesy et al. 2025). Older people are more likely to get the infection because they may have other health problems, weaker immune systems, take many medications, not eat well, and have less physical strength as they age. The infection rate among the healthy < 15-year-old group was the lowest (4.5%) compared to other age groups. These may be observed as a result of a healthy immune system at an earlier age, as well as a reduction in the ingestion of raw meat, street food, and travel hazards as people age. This conclusion was in line with Farag et al. (2023) and Zhou et al. (2021).

Regarding residence, the outcomes revealed that the occurrence of *E. coli* and *E. coli* O157:H7 was insignificantly higher in rural areas than in urban areas among diarrhoeal and healthy individuals. This finding suggests that individuals residing in rural areas are at a higher risk of contracting the infection than those in urban areas. This result matches Byrne et al. (2015), who indicated that animals and environmental factors pose a significant risk of disease spreading, whether through direct contact or indirect means, such as instruments contaminated with the faeces of farm animals that are reservoirs for *E. coli*. The current findings agree with earlier studies that showed higher rates of infection in rural areas compared to urban areas, with rates of 55.17% and 38.09% (Farag et al. 2023) and 46.58% and 21.11% (Zhou et al. 2021). This study reveals similar levels of *E. coli* and *E. coli* O157:H7 in food, animals, and human specimens, suggesting potential hazard aspects, such as similar environmental circumstances or pathways for bacterial transmission (Zhou et al. 2021).

The O serogroup of *E. coli* is a key predictor of its pathology. Hence, it can reveal crucial details on the pathogenicity of *E. coli* strains (Elbastawesy et al. 2025). Therefore, we subjected the *E. coli* strains to O genotyping. The strains examined in this study exhibited a wide range of O-genotypes, including several that are clinically relevant. The *E. coli* strains studied were found to belong to the serogroups O157, O55, O86, O114, O126, O158, O26, O78, O119, O121, O125, and O142. These findings indicate the identification of various serogroups in food sources, as well as their association with diarrhoeic patients. The study highlighted that food samples that contain these serotypes may be one of the potential causes of *E. coli* diarrhoea and have been linked to outbreaks and certain human illnesses (Dandrawy et al. 2025). Animal-derived foods or plants contaminated with these animals’ faeces are associated with human illnesses induced by *E. coli* O157:H7. Particularly cattle, sheep, and goats have been shown as the primary natural reservoirs for *E. coli* O157:H7 and are of significant public health relevance (Tayh et al. 2022).

The results of this investigation match those of Dandrawy et al. (2025), who found that the most common serotypes in meat products were O158, O142, O63, O119, O55, O169, O124, O114, O27, O127, O111, O78, O86, O146, O26, and O153. Another study by Younis et al. (2021) revealed that O111, O27, O114, and O125 were the most identified serotypes in raw milk. Tayh et al. (2022) verified all isolates from cattle faecal samples as *E. coli* O157:H7. Farag et al. (2023) revealed different types of *E. coli* from numerous sources: O26, O104, O126, O44, O145, and O103 in raw milk; O86, O121, O157, O119, O142, and O128 in karish cheese; O86, O118, O26, O126, O114, O125, O103, and O145 in minced meat; and O45, O121, O26, O103, O145, O111, and O118 in patients with diarrhoea. Variation in serotype detection may be due to differences in the environmental spreading of STEC strains and sampling methods.

Molecular approaches are more precise for making conclusive identifications. The housekeeping gene *O157* and *fliCH7* of *E. coli* O157:H7, a molecular diagnostic marker, were positive in all detected phenotypic and serological *E. coli* O157:H7 isolates. The public health significance of *E. coli* O157:H7 strains as a reason for severe food illness in humans is highlighted by the fact that all of the strains obtained in this research inherited one or more enterotoxigenic genes. This matches with earlier studies by Altaie et al. (2025), Dandrawy et al. (2025), Farag et al. (2023), and Tayh et al. (2022).

The main virulence markers of *E. coli* O157:H7 are Shiga toxin (*Stx1* and *Stx2*) genes, which are commonly linked to severe diseases such as hemolytic-uremic syndrome and hemorrhagic colitis (Tayh et al. 2022). Although both toxin types share the same cell receptor and are structurally similar, *Stx2* is substantially more toxic than *Stx1*, as it exhibits higher affinity for host cell ribosomes and displays greater catalytic activity when assessed in cytotoxicity tests using Vero and HeLa cells (Dandrawy et al. 2025). Reflecting this, certain *Stx* subtypes, including *stx*_*1a*_, *stx*_*2a*_, *stx*_*2b*_, *stx*_*2c*_, and *stx*_*2d*_, are more commonly associated with human disease than others, while the different subtypes also appear to vary in the severity of diseases that they are associated with (Tayh et al. 2022). The *eae* gene produces an intimin protein necessary for adhering and destroying activity in host intestinal cells and is responsible for serious human disorders. Additionally, the *hlyA* gene encodes enterohemolysin, which lyses erythrocytes and contributes to intestinal iron absorption. This gene is widely utilized as an epidemiological indicator for STEC strains (Tayh et al. 2022). Plasmids transmit the *hlyA* gene, facilitating its transfer between bacterial isolates (Altaie et al. 2025).

In this study, the most frequently found virulence genes in *E. coli* O157:H7 were *Stx1* (42.4%) and *uidA* (39.4%), followed by *eaeA* (36.4%), *hlyA* (30.3%), and *Stx2* (15.2%). Some samples from both humans and food showed the same virulence gene pattern, demonstrating that *E. coli* O157:H7 could be a serious public health risk (Khairy et al. 2024). This observation matches the outcomes of Altaie et al. (2025), who documented that all *E. coli* O157:H7 isolates from food, animal, and human samples harboured one or more virulence genes (*Stx1*, *Stx2*, *eae*, and *hlyA*), and Bumunang et al. (2022), who showed that the *E. coli* O157:H7 obtained from clinical isolates carried *Stx1* and *Stx2*. A study by Tayh et al. (2022) found that all strains had the *uidA*, *flicH7*, and *O157* genes; 70% had the *Stx2* and *hlyA* genes; and 60% had the *Stx1* and *eae* genes, indicating a possible connection to the host. Additionally, the PCR results from Farag et al. (2023) showed that the most common genes found in the tested food and human samples were *eaeA* (90%), then *Stx2* (30%), *hlyA* (30%), and *Stx1* (10%).

The study emphasizes the serious epidemiological concern of antimicrobial resistance (AMR) in *E. coli* O157:H7 isolates from food products, animals, and humans. Antimicrobial resistance has been proven as a growing international issue, and antibiotic-resistant *E. coli* O157:H7 has been reported in numerous studies. In this study, most *E. coli* O157:H7 strains were completely resistant to ampicillin, penicillin G, and tetracycline. At the same time, they were fully sensitive to florfenicol and ciprofloxacin, making those the most important antibiotics. The *E. coli* O157:H7 strain was resistant to multiple drugs, with resistance levels ranging from 0.200 to 0.867 and an average of 0.567, indicating that these strains likely came from high-risk contamination (> 0.2) (El-Hawary et al. 2024). Also, the intermediate susceptibility profiles and resistance reports are important because they show that the organism may be becoming resistant. These results match findings from other studies by Ababu et al. (2020), Altaie et al. (2025), Omara et al. (2024), Tayh et al. (2022), and Younis et al. (2021), which also found that bacteria were significantly resistant to antibiotics. The differences in antimicrobial resistance could be caused by the presence of resistance genes in bacteria found in animals, the environment, or humans, and these differences in resistance levels might also reflect how antibiotics and antimicrobials are used in farming and animal care (Elbarbary et al. 2025).

In this investigation, some bacteria were resistant to the antibiotics tested because they had one or more antibiotic-resistant genes, like *tetA*, *blaTEM*, and *Aada1*. These findings are matched with the outcomes of Omara et al. (2024), Tayh et al. (2022), and Younis et al. (2021). They identified the *tetA* gene that causes resistance to tetracycline, the *Aada1* gene that causes resistance to aminoglycosides, and the *blaTEM* gene that causes resistance to beta-lactam antibiotics (Younis et al. 2021). The results emphasize the hazards of overusing antibiotics, as the practice encourages the development and transmission of resistant strains via direct contact or the food chain. The One Health policy is critical for reducing MDR *E. coli* O157:H7 hazards while also preserving public health and animal welfare (Zhou et al. 2021).

The food industry is currently confronted with the challenge of addressing consumers’ concerns regarding the quality, safety, and conservation of food on a global scale (Elbarbary et al. 2023). This goal is achieved by limiting the use of artificial chemical additives. The viability of employing natural antioxidants to maintain and enhance the general quality of food products has been assessed utilizing a broad range of plant products (Alqdeeri et al. 2020). Extracts from several plant portions, such as the roots, stems, flowers, and fruits, are crucial for stopping microbial growth and spoilage (Rybczy et al. 2023). This idea suggests that using different compounds, like PcL, could help identify better and safer preservatives than those we usually use. PcL and its constituents have many health benefits, such as reducing inflammation, lowering blood sugar, anticancer, protecting the kidneys and liver, boosting the immune system, antidepressant, antibacterial, antiparasitic, repelling insects, helping wounds heal, and reducing anxiety, as shown by many studies (Alqadeeri et al. 2020; Drissi et al. 2022). Additionally, PcL shows strong antioxidant properties compared to synthetic antioxidants and has antibacterial effects against many foodborne pathogens, which makes it a promising natural food preservative (Rahman et al. 2023).

In the current experiment, the antibacterial property of the PcL extract was determined by analyzing the extract’s ability to control the growth of harmful *E. coli* O157:H7 using the disc diffusion approach. All concentrations of PcL extracts showed inhibitory zones against *E. coli* O157:H7 (*p* < 0.05). The inhibitory zone diameter varied from 5.2 ± 0.7 mm at 0.5% PcL to 17.7 ± 0.5 mm at 5% PcL concentration. This investigation agrees with the results of Alqadeeri et al. (2020), Fazly Ann et al. (2019), and Rahman et al. (2023). This research supports the common use of Piper plants, particularly Piper cubeba, as they show promise in providing new treatments against foodborne pathogens and dangerous microorganisms affecting humans.

The MIC is considered the benchmark for evaluating the susceptibility of microbes to antibiotics. It is described as the antimicrobial agent’s lowest concentration or greatest dilution that, following an overnight incubation period, visibly suppresses the development of bacteria. The MBC was defined as the lowest dose of an antimicrobial agent that inhibited microorganism growth (Fazly Ann et al. 2019). The MIC data shown here indicate that the PcL extract was very effective at killing the *E. coli* O157:H7 strain, with MICs from 0.43 to 0.74 mg/ml. In the study of the PcL extract as a bactericidal agent, some isolates had an MBC rate that was similar to the MIC rate. Statistically, there was a significant difference between the MIC and MBC values of the extract and the control (*p* < 0.05). The results indicated that the antibacterial impact of the PcL extract employed in this study was just as effective as the commercial antibiotic against the examined *E. coli* O157:H7. These outcomes were matched with those of earlier research by Fazly Ann et al. (2019) and Gomez-Llorente et al. (2025).

The antibacterial effect of PcL is attributed to the presence of key chemicals, including polyphenolics and flavonoids (such as rutin, caffeic acid, gallic acid, catechin, and ferulic acid), lignans (like hinokinin, Cubebininolide, isoyatein, and yatein), fatty acids (including lauric acid, palmitic acid, hexadecanoic acid, and 9-octadecenoic acid), and fragrant substances (such as eugenol, β-cubebene, and α-cubebene) (Drissi et al. 2022). It is known that phenolic compounds have an inhibitory effect on hydrogen peroxide-induced lipid peroxidation in biological homogenates. The redox characteristics of phenolic compounds, which can be useful in adsorbing and neutralizing superoxide anion (O₂⁻), hydroxyl radical, or peroxy radicals, quenching singlet and triplet oxygen, or dissolving peroxides, are primarily responsible for their antioxidant action. By converting free radicals and reactive oxygen species into more stable products, phenolic contents seem to serve as potent electron and hydrogen atom donors, interrupting the radical chain reaction that leads to lipid peroxidation (Rahman et al. 2023).

Time-kill curves are commonly employed to assess the efficacy of antimicrobials by tracking the growth and death of bacteria across various concentrations (Foerster et al. 2016). In this study, the time-kill curve of the PcL extract against *E. coli* O157:H7 was plotted, showing that the number of bacteria in the sample not treated with PcL extract (0.0 × MIC) grew significantly from 0 to 12 h. After 12 h of incubation, samples treated with 1 × MIC (0.43 mg/mL) PcL extract showed a modest log reduction. However, the concentration was insufficient to inhibit bacterial growth. The bacterial count decreased following the treatments of 2 x MIC (0.86 mg/mL), 3 x MIC (1.29 mg/mL), and 4 x MIC (1.72 mg/mL). The results indicated that the bactericidal activity of PcL extract against *E. coli* O157:H7 was expressed after 12 h at a concentration of 1.29 mg/mL and after 8 h at a concentration of 1.72 mg/mL (*p* < 0.05). This conclusion is in line with earlier studies (Fazly Ann et al. 2019; Gomez-Llorente et al. 2025). This work demonstrates that the reduction in bacterial count is closely associated with increasing extract concentration and treatment period. This decrease might be owing to the bacteria’s great susceptibility to the polyphenols present in abundance in this extract, such as CA, vanillin, and CH. Due to vanillin’s hydrophobic nature, the majority of its antimicrobial mechanisms are based solely on its capacity to damage the cytoplasmic membrane of microorganisms through interactions with lipids, proteins, or both structures, resulting in the depletion of the ionic gradientand the suppression of oxidative metabolism (Rahman et al. 2023).

The study has some limitations, such as: (1) Due to the limited time range of the sampling, the results may not be representative of the true seasonality of *E. coli* prevalence and resistance trends. Environmental conditions, like temperature, water flow, and agricultural practices, affect bacterial load and diversity. (2) Human isolates came from a certain group of people, which could lead to selection bias. The statistical validity of the human information was restricted by the absence of systematic control over variations in health status, age, healthcare access, or exposure hazards. (3) The study design was cross-sectional, which meant that it only provided a snapshot of the distribution of *E. coli* and antibiotic resistance at a single point. There was no longitudinal follow-up data collected. (4) We didn’t perform whole genome sequencing or comparative analyses of the bacterial isolates derived from various sources to ascertain whether they were genetically distinct or genetically identical. However, they were contaminated with different samples. These limitations make it clear that more study is needed with samples taken all year, human groups that are more diverse and representative, and long-term monitoring to learn more about how *E. coli* spreads and how it affects people in the region. Furthermore, additional research should focus on the quality control of natural preservatives because of inconclusive information regarding their safety and toxicity.

## Conclusion

The current findings highlight the serious threat that *E. coli* O157:H7, which carries one or more virulence and antibiotic-resistant genes, poses to public health in humans and animals. Antibiotic resistance calls for more monitoring, stricter food safety standards, and reasonable antibiotic use in humans and animals. These results emphasize the value of continuous monitoring and public health initiatives that minimize the prevalence of illnesses linked to *E. coli* and improve food safety in Egypt. Using *Piper cubeba* extracts as a natural antibacterial could be a useful way to reduce the chances of getting sick from *E. coli* O157:H7 and its presence in food, whether at home or in public places, suggesting it might be a beneficial natural substitute for regular preservatives. Adequate hygienic measures during the food processing and distribution stages of the food chain, as well as proper animal handling techniques on farms and slaughterhouses, can reduce the risk of food contamination and infection for individuals working with animals. A future study of the effects of antibacterial agents on various strains and their antibacterial mechanisms of action is required to advance in this sector. Furthermore, it is critical to identify the ideal dosages and combinations of diverse natural antimicrobial agents for food preservation to examine their potential synergistic effects.

## Supplementary Information

Below is the link to the electronic supplementary material.

Supplementary Material 1 (DOCX 30.5 KB)

Supplementary Material 2 (DOCX 1.09 MB)

## Data Availability

The manuscript included all our available data. All data related to Figs. 1, 5 and 6, and 7 were available and visualized using online software using a heatmap (heatmap v7.0.1, [https://app.flourish.studio/visualisation/23052065/edit](https:/app.flourish.studio/visualisation/23052065/edit)) and hierarchical bars (Hierarchy v24.1.1, [https://app.flourish.studio/visualisation/23054964/edit](https:/app.flourish.studio/visualisation/23054964/edit)).

## Declarations

### Guidelines

All methods were carried out in accordance with relevant guidelines and regulations.
