# Supplementary material for: Escherichia coli O157:H7 prevalence in Upper Egypt: impacts on food safety and human Health, with a protection trial using natural antibacterial Piper cubeba
Source: World J Microbiol Biotechnol. 2025 Nov 13;41(11):453. doi: 10.1007/s11274-025-04620-3 (PMC12615562; doi:10.1007/s11274-025-04620-3)
Supplement: Supplementary file 1 — Supplementary Material 1 (DOCX 30.5 KB) [file 11274_2025_4620_MOESM1_ESM.docx]

**Supplementary Tables (S)**

**Table S1: Serotyping of *E. coli* Strains isolated from different sources (*n*=115)**

| **Source** | **No.** | ***E. coli* serotypes no. (%)** | | | | | | | | | | | |
| --- | --- | --- | --- | --- | --- | --- | --- | --- | --- | --- | --- | --- | --- |
|  |  | **O126** | **O114** | **O55** | **O26** | **O86** | **O78** | **O157** | **O125** | **O119** | **O158** | **O142** | **O121** |
| Minced beef | 5 | 1  (20) | 1  (20) | 0 | 1  (20) | 0 | 0 | 1  (20) | 0 | 1  (20) | 0 | 0 | 0 |
| Beef kofta | 6 | 0 | 2 (33.3) | 1 (16.7) | 0 | 0 | 1  (16.7) | 2  (33.3) | 0 | 0 | 0 | 0 | 0 |
| Beef burger | 6 | 0 | 1 (16.7) | 0 | 0 | 0 | 2  (33.3) | 2  (33.3) | 0 | 0 | 1 (16.7) | 0 | 0 |
| Raw cow milk | 5 | 1  (20) | 0 | 0 | 1  (20) | 0 | 0 | 2  (40) | 1  (20) | 0 | 0 | 0 | 0 |
| Karish cheese | 4 | 0 | 0 | 1  (25) | 0 | 0 | 0 | 1  (25) | 0 | 0 | 0 | 0 | 2  (50) |
| Yoghurt | 3 | 1 (33.3) | 0 |  | 0 | 0 | 0 | 1 (33.3) | 1 (33.3) | 0 | 0 | 0 | 0 |
| Ox | 7 | 1 (14.3) | 0 | 2 (28.6) | 0 | 0 | 2  (28.6) | 1 (14.3) | 0 | 1 (14.3) | 0 | 0 | 0 |
| Cow | 8 | 0 | 1 (12.5) | 0 | 2  (25) | 2  (25) | 0 | 3 (37.5) | 0 | 0 | 0 | 0 | 0 |
| Sheep | 6 | 1 (16.7) | 0 | 2 (33.3) | 0 | 0 | 0 | 1 (16.7) | 0 | 0 | 0 | 1  (16.7) | 1  (16.7) |
| Goat | 4 | 0 | 1  (25) | 0 | 0 | 2  (50) | 0 | 1  (25) | 0 | 0 | 0 | 0 | 0 |
| Nasser Lake | 7 | 2 (28.6) | 0 | 0 | 0 | 1 (14.3) | 0 | 2 (28.6) | 0 | 0 | 1 (14.3) | 0 | 1  (14.3) |
| River Nile | 9 | 1 (11.1) | 1 (11.1) | 2 (22.2) | 0 | 0 | 0 | 3 (33.3) | 0 | 2 (22.2) | 0 | 0 | 0 |
| Diarrheic | 38 | 0 | 2  (5.3) | 10 (26.3) | 3 (7.9) | 6 (15.8) | 0 | 10 (26.3) | 0 | 1  (2.6) | 5 (13.2) | 1 (2.6) | 0 |
| Apparently healthy | 7 | 0 | 0 | 2 (28.6) | 0 | 0 | 0 | 3 (42.9) | 1 (14.3) | 0 | 1  (14.3) | 0 | 0 |
| **Total no. (%)** | 115 | 8  (7.0) | 9  (7.8) | 20  (17.4) | 7  (5.2) | 11  (9.6) | 5  (4.3) | 33  (28.7) | 3  (2.6) | 5  (4.3) | 8 (7.0) | 2  (1.7) | 4  (3.4) |

**Table S2: Distribution of virulence and antibiotic resistance genes in *E. coli* O157: H7 strains screened by PCR**

| **Source** | **No.** | **Virulence genes** | | | | | | | **Antibiotic resistance genes** | | |
| --- | --- | --- | --- | --- | --- | --- | --- | --- | --- | --- | --- |
|  |  | ***O157*** | ***fliCH7*** | ***uidA*** | ***stx1*** | ***stx2*** | ***eaeA*** | ***hlyA*** | ***tetA*** | ***blaTEM*** | ***Aada1*** |
| Minced beef | 1 | 1 | 1 | 0 | 1 | 0 | 0 | 0 | 0 | 1 | 0 |
| Beef kofta | 2 | 2 | 2 | 1 | 0 | 1 | 0 | 2 | 2 | 0 | 0 |
| Beef burger | 2 | 2 | 2 | 2 | 1 | 0 | 1 | 0 | 1 | 1 | 0 |
| Raw cow milk | 2 | 2 | 2 | 0 | 2 | 1 | 1 | 0 | 0 | 1 | 2 |
| Karish cheese | 1 | 1 | 1 | 0 | 1 | 0 | 0 | 1 | 1 | 0 | 0 |
| Yoghurt | 1 | 1 | 1 | 0 | 0 | 0 | 1 | 0 | 0 | 1 | 0 |
| Ox | 1 | 1 | 1 | 1 | 0 | 0 | 0 | 0 | 1 | 0 | 1 |
| Cow | 3 | 3 | 3 | 2 | 2 | 1 | 0 | 1 | 1 | 2 | 1 |
| Sheep | 1 | 1 | 1 | 0 | 0 | 0 | 1 | 1 | 1 | 0 | 0 |
| Goat | 1 | 1 | 1 | 0 | 1 | 0 | 0 | 1 | 0 | 0 | 0 |
| Nasser Lake | 2 | 2 | 2 | 1 | 0 | 0 | 2 | 0 | 0 | 0 | 0 |
| River Nile | 3 | 3 | 3 | 1 | 2 | 0 | 0 | 2 | 1 | 1 | 0 |
| Diarrheic | 10 | 10 | 10 | 4 | 3 | 2 | 4 | 2 | 3 | 6 | 4 |
| Apparently healthy | 3 | 3 | 3 | 1 | 1 | 0 | 2 | 0 | 1 | 1 | 1 |
| Total no. (%) | 33 | 33  (100) | 33  (100) | 13  (39.4) | 14  (42.4) | 5  (15.2) | 12  (36.4) | 10  (30.3) | 12  (36.4) | 14  (42.4) | 9  (27.3) |

**Table S3: Antimicrobial pattern of *E. coli* O157: H7 isolates (*n*=33)**

| **Antimicrobial agents** | **Sensitive** | | **Intermediate** | | **Resistance** | |
| --- | --- | --- | --- | --- | --- | --- |
|  | **No.** | **%** | **No.** | **%** | **No.** | **%** |
| GT | 19 | 57.6 | 6 | 18.2 | 8 | 24.2 |
| KAN | 14 | 42.4 | 3 | 9.1 | 16 | 48.5 |
| CPL | 20 | 60.6 | 5 | 15.2 | 8 | 24.2 |
| FLO | 33 | 100 | 0 | 0 | 0 | 0 |
| AMP | 0 | 0.0 | 0 | 0 | 33 | 100 |
| PEN | 0 | 0.0 | 0 | 0 | 33 | 100 |
| CIP | 33 | 100 | 0 | 0 | 0 | 0 |
| ENR | 31 | 94.0 | 2 | 6.1 | 0 | 0 |
| PB | 17 | 52.0 | 7 | 12.2 | 9 | 27.3 |
| SLT | 3 | 9.1 | 7 | 12.2 | 23 | 69.7 |
| TET | 0 | 0.0 | 0 | 0 | 33 | 100 |
| EM | 0 | 0.0 | 6 | 18.2 | 27 | 82.0 |
| AZM | 13 | 39.4 | 0 | 0 | 20 | 60.6 |
| NAL | 17 | 51.5 | 3 | 9.1 | 13 | 39.4 |
| RIF | 27 | 81.8 | 0 | 0 | 6 | 18.2 |
| *p* value | *p* < 0.0014 | | *p* < 0.0001 | | *p* < 0.0001 | |

Gentamycin (GT), kanamycin (KAN), chloramphenicol (CPL), florfenicol (FLO), ampicillin (AMP), penicillin G (PEN), ciprofloxacin (CIP), enrofloxacin (ENR), polymyxin B (PB), sulfamethoxazole-trimethoprim (SLT), tetracycline (TET), erythromycin (EM), azithromycin (AZM), nalidixic acid (NAL), rifampin (RIF).

**Table S4: Antibiogram profile of *E. coli* O157: H7 isolates (*n* = 33)**

| **source** | **Isolates no.** | **Antimicrobial resistance profile** | **No. of antibiotic** | **MAR index** |
| --- | --- | --- | --- | --- |
| Kofta, burger, raw milk, cow, Nasser Lake, River Nile, diarrheic | 11 | GT, KAN, CPL, AMP, PEN, ENR, PB, SLT, TET, EM, AZM, NAL, RIF | 13 | 0.867 |
| Minced beef, burger, karish cheese, River Nile, diarrheic, apparently healthy | 9 | GT, KAN, CPL, AMP, PEN, PB, SLT, TET, EM, AZM, NAL | 11 | 0.733 |
| kofta Sheep, Goat, Nasser Lake, diarrheic, apparently healthy | 6 | GT, KAN, AMP, PEN, PB, SLT, TET, EM, AZM | 9 | 0.600 |
| Raw milk, yoghurt, ox, diarrheic | 3 | KAN, AMP, PEN, PB, SLT, TET, EM, NAL | 8 | 0.533 |
| River Nile, diarrheic apparently healthy | 2 | CPL, AMP, PEN, PB, SLT, TET, EM | 7 | 0.467 |
| Diarrheic | 2 | AMP, PEN, TET | 3 | 0.200 |
|  | 33 | MAR average |  | 0.567 |

MAR: multiple antibiotics resistant. Gentamycin (GT), kanamycin (KAN), chloramphenicol (CPL), florfenicol (FLO), ampicillin (AMP), penicillin G (PEN), Ciprofloxacin (CIP), enrofloxacin (ENR), polymyxin B (PB), sulfamethoxazole-trimethoprim (SLT), tetracycline (TET), erythromycin (EM), azithromycin (AZM), nalidixic acid (NAL), rifampin (RIF).

**Table S5: Time-kill curve plot of *E. coli* O157: H7 after different exposure time to different *PcL* concentrations**

| ***PcL*** **concentrations (%)** | **Exposure time (h) / log_10_** | | | | |
| --- | --- | --- | --- | --- | --- |
|  | 0 | 1 | 4 | 8 | 12 |
| 0 × MIC | 6.7 | 6.8 | 7.9 | 9.7 | 10.9 |
| 1 × MIC | 6.7 | 6.4 | 5.8 | 5.3 | 4.9 |
| 2 × MIC | 6.7 | 4.8 | 4.2 | 3.7 | 1.6 |
| 3 × MIC | 6.7 | 3.6 | 2.2 | 1.4 | 0 |
| 4 × MIC | 6.7 | 2.4 | 1.3 | 0 | 0 |
