# Supplementary figures and images for: Escherichia coli O157:H7 prevalence in Upper Egypt: impacts on food safety and human Health, with a protection trial using natural antibacterial Piper cubeba
Source: World J Microbiol Biotechnol. 2025 Nov 13;41(11):453. doi: 10.1007/s11274-025-04620-3 (PMC12615562; doi:10.1007/s11274-025-04620-3)

**Supplementary file**

**Fig. 2**

**
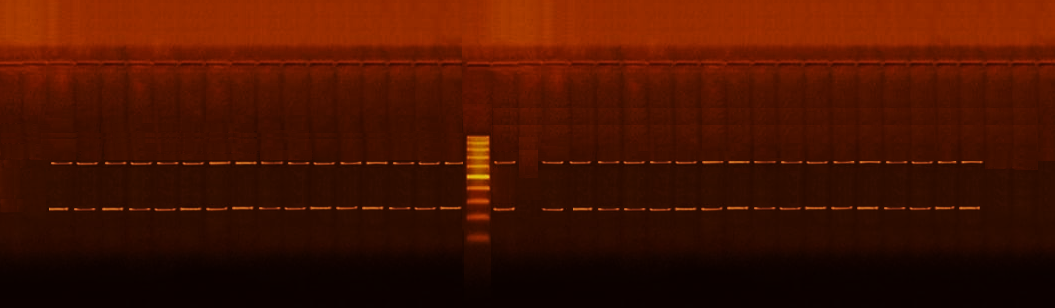
**

**Fig. 3**

**
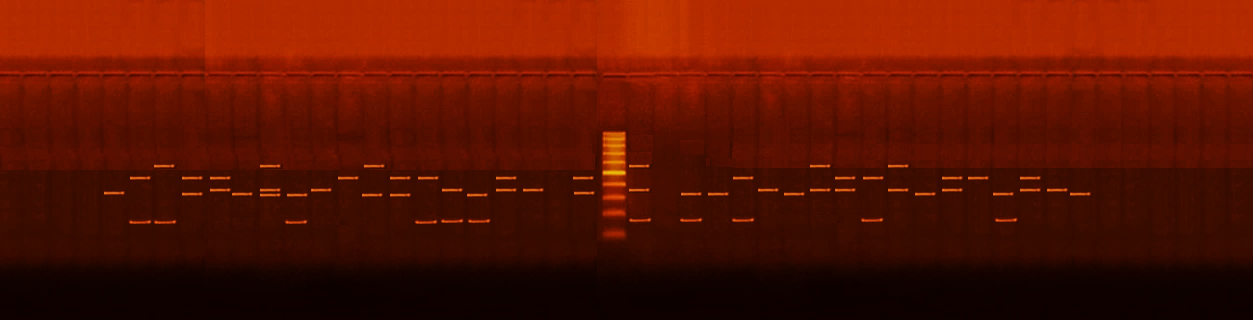
**

**Fig. 4**

**
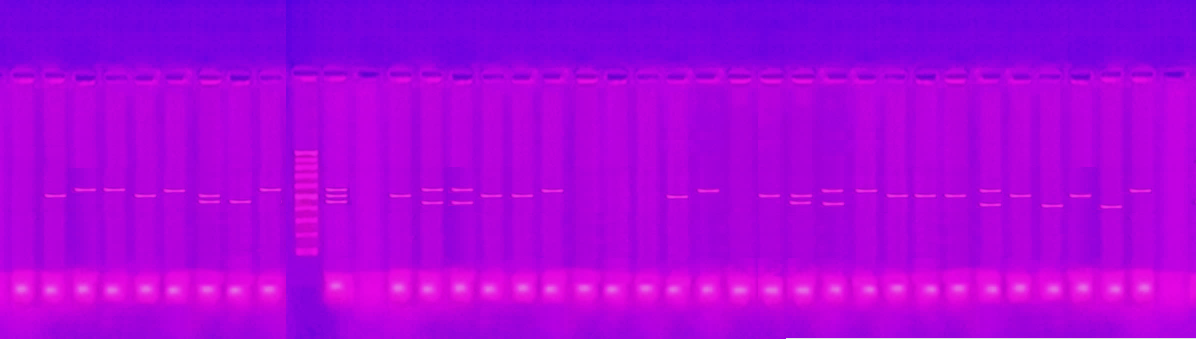
**

Supplement: Supplementary file 2 — Supplementary Material 2 (DOCX 1.09 MB) [file 11274_2025_4620_MOESM2_ESM.docx]
